# Efficient ammonium uptake and mobilization of vacuolar arginine by *Saccharomyces cerevisiae* wine strains during wine fermentation

**DOI:** 10.1186/s12934-014-0109-0

**Published:** 2014-08-19

**Authors:** Lucie Crépin, Isabelle Sanchez, Thibault Nidelet, Sylvie Dequin, Carole Camarasa

**Affiliations:** INRA, UMR1083 Sciences Pour l’Œnologie 2 Place Viala, Montpellier, F-34060 France; SupAgro, UMR1083 Sciences Pour l’Œnologie 2 Place Viala, Montpellier, F-34060 France; Université Montpellier I, UMR1083, Sciences Pour l’Œnologie 2 Place Viala, Montpellier, F-34060 France

**Keywords:** Diversity, *Saccharomyces cerevisiae*, Nitrogen, Biomass production, Wine fermentation

## Abstract

**Background:**

Under N-limiting conditions, *Saccharomyces cerevisiae* strains display a substantial variability in their biomass yield from consumed nitrogen -in particular wine yeasts exhibit high growth abilities- that is correlated with their capacity to complete alcoholic fermentation, a trait of interest for fermented beverages industries. The aim of the present work was to assess the contribution of nitrogen availability to the strain-specific differences in the ability to efficiently use N-resource for growth and to identify the underlying mechanisms. We compared the profiles of assimilation of several nitrogen sources (mostly ammonium, glutamine, and arginine) for high and low biomass-producing strains in various conditions of nitrogen availability. We also analyzed the intracellular fate of nitrogen compounds.

**Results:**

Strains clustered into two groups at initial nitrogen concentrations between 85 and 385 mg N.L^−1^: high biomass producers that included wine strains, were able to complete fermentation of 240 g.L^−1^ glucose and quickly consume nitrogen, in contrast to low biomass producers. The two classes of strains exhibited distinctive characteristics that contributed to their differential capacity to produce biomass. The contribution of each characteristic varied according to nitrogen availability. In high biomass producers, the high rate of ammonium uptake resulted in an important consumption of this preferred nitrogen source that promoted the growth of these yeasts when nitrogen was provided in excess. Both classes of yeast accumulated poor nitrogen sources, mostly arginine, in vacuoles during the first stages of growth. However, at end of the growth phase when nitrogen had become limiting, high biomass producers more efficiently used this vacuolar nitrogen fraction for protein synthesis and further biomass formation than low biomass producers.

**Conclusions:**

Overall, we demonstrate that the efficient management of the nitrogen resource, including efficient ammonium uptake and efficient use of the amino acids stored in vacuoles during the late stages of growth, might lead to high biomass production by wine yeasts.

**Electronic supplementary material:**

The online version of this article (doi:10.1186/s12934-014-0109-0) contains supplementary material, which is available to authorized users.

## Background

Nitrogen is the limiting nutrient for yeast growth in most grape juice fermentations. Consequently, the amount of biomass that is produced is correlated with the initial nitrogen concentration of grape must, and this relationship is strongest when nitrogen is present in the medium at concentrations lower than 300 mg N.L^−1^ [1–5]. Nitrogen limitation is one of the main causes of stuck fermentation [2,6,7], which is probably a consequence of insufficient biomass yield rather than a low metabolic activity of cells [8,9]. Thus, an increase in nitrogen availability in nitrogen-limited wine must results in both a higher fermentation rate and a decrease in the total fermentation time [2,10,11]. It is commonly accepted that the minimal concentration of nitrogen required for completion of alcoholic fermentation by *S. cerevisiae* strains is around 140 mg N.L^−1^ [1,2,12]. However, this threshold value is strain-dependent, and nitrogen demands, defined as the amount of ammoniacal nitrogen necessary to maintain a constant rate of fermentation during the stationary phase and as the minimum quantity required to ensure the maximum population during growth, differ substantially between strains [13–15].

In line with these observations, a population of 72 *S. cerevisiae* strains originating from a wide range of habitats shows a substantial diversity in biomass yield from nitrogen consumed during alcoholic fermentation [16]. Yeasts with low biomass production are also unable to complete the fermentation of 240 g.L^−1^ sugar. Interestingly, all wine yeasts (9 strains) are high biomass producing strains and the dispersion (standard deviation) within this group of phenotypic variables in line with growth and fermentative activity is substantially lower than that of the whole population [16]. We previously examined the nitrogen assimilation profile of 14 strains in the presence of 165 mgN.L^−1^ yeast assimilable nitrogen (YAN) [17]. Yeast showed substantial variability in their kinetics of nitrogen uptake, with low biomass-producing strains displaying a low rate of nitrogen assimilation. However, little is known about the mechanisms underlying these dissimilarities between strains. YAN in grape must is a complex mixture of ammonium ions and amino acids at various concentrations [12]. The order of nitrogen sources consumption is similar amongst strains [17]; therefore, variations in biomass yield may result from differences in the rate of uptake of YAN as a whole, or in the ability of a strain to efficiently assimilate particular nitrogen compounds. Alternatively, the fate of a nitrogen source may depend on the particular strain that consumes it. Internalized nitrogen compounds can be allocated in diverse ways, according to the anabolic requirements of the cells [18,19]. Amino acids may be used directly in biosynthetic processes, and incorporated into proteins; other fractions may provide amine groups, or may be de-aminated to generate ammonia, or may serve as substrates for transaminases. Assimilation of ammonium, together with amino acid transamination reactions, results in the formation of glutamate from α-ketoglutarate, which can be converted in turn into glutamine [18,19]. Glutamine and glutamate are central to the *de novo* synthesis of amino acids. They are used by carbon precursors (mainly α-keto acids) derived from the central carbon metabolism as donors of –NH_3_ groups (85% from glutamate and 15% from glutamine). In addition, a proportion of amino acids can accumulate in vacuoles. This storage allows the cytosolic pool of amino acids to be regulated for efficient protein synthesis [20]. Transport into the vacuolar compartment depends on the nature of the nitrogen compound: a large fraction of basic amino acids is compartmentalized in vacuoles, whereas some amino acids, particularly glutamate and aspartate, are found exclusively in the cytoplasm [20].

Biomass production is an important issue for fermentation; therefore, an understanding of the mechanisms responsible for the strain-specific differences in biomass production from consumed nitrogen during wine fermentation would be valuable. We previously observed differences in YAN consumption between high and low biomass-producing yeasts under N-limiting conditions [17]. The first objective of this study was to investigate whether these differences are influenced by nitrogen availability in the medium. We also aimed to identify the mechanisms underlying these dissimilarities that may explain the high biomass formation by wine yeasts. We examined the uptake of the diverse nitrogen sources contained in grape juice by the two classes of strains and assessed the fate of these molecules inside the cells. We particularly focused on the most abundant nitrogen compounds, namely arginine, ammonium and glutamine.

## Materials and methods

### Yeast strains

The *Saccharomyces cerevisiae* strains used in this study were selected based on their biomass yield from nitrogen consumed under enological conditions [16]. They were collected from natural environment (oak bark) and industrial processes and were obtained from the Sanger Institute and various companies (Table 1).

**Table 1:** **Origins and sources of** ***S. cerevisiae*** **strains studied**

**Environment**	**Strain**	**Geographical origin**	**Origin**
Industrial processes			
Palm winemaking	NCYC110	Nigeria, West Africa	Sanger
Commercial winemaking	EC1118	France	Lalvin
	L2226	France	Enoferm
	WE372	South Africa	Anchor
Natural environment			
Oak	YPS128	Pennsylvania, USA	Sanger
	YPS1009	New Jersey, USA	Washington

### Fermentation conditions

An aliquot of a reference stock (conserved at -80°C) was transferred to a YPD agar plate (1% Bacto yeast extract, 2% bactopeptone, 2% glucose, 1.5% agar) for each strain 48 h before fermentation. Initial cultures were grown in YPD medium in 10 mL flasks at 28°C with shaking (150 rpm) for 12 h. Cultures were then transferred to 10 mL flasks containing synthetic medium (SM) supplemented with 165 mgN.L^−1^ and were grown for 12 h at 28°C with shaking (150 rpm). These pre-cultures were used to inoculate fermentations (10^6^ cells.L^−1^) in SM containing 240 g.L^−1^ glucose, 6 g.L^−1^ malic acid, 6 g.L^−1^ citric acid, and 200 mg.L^−1^ nitrogen at pH 3.5 [2]. Ergosterol (1.875 mg.L^−1^), oleic acid (0.625 mg.L^−1^) and Tween 80 (0.05 g.L^−1^) were provided as anaerobic growth factors. These fermentations were performed in 1 L fermentors equipped with fermentation locks to maintain anaerobiosis, at 28°C with continuous magnetic stirring (150 rpm).

Nitrogen was supplied as a mixture of amino acids and NH_4_Cl mimicking the typical composition of grape juice. Several media with different initial nitrogen concentrations were used (SM45: 45 mgN.L^−1^, SM85: 85 mgN.L^−1^, SM165: 165 mgN.L^−1^, SM260: 260 mgN.L^−1^ and SM385: 385 mgN.L^−1^) (Additional file 1: Table S1).

### Analytic methods

CO_2_ release was determined by automatic measurements of fermentor weight every 20 min. The rate of CO_2_ production, (dCO_2_/dt) is the first derivative of the amount of CO_2_ produced over time and was calculated automatically by polynomial smoothing of the CO_2_ production curve [21].

The population size was monitored by counting cells with an electronic particle counter (Multisizer 3 Coulter Counter, Beckman Coulter) fitted with a probe with a 100 mm aperture. Dry weight was determined by filtering 50 mL of culture though a 0.45 μm pore-sized nitrocellulose filter (Millipore), which was washed twice with 50 mL of distilled water and dried for 48 h at 105°C.

The ammonium ion concentration in the supernatant (sample centrifugation, 3 000 g, 4°C, 10 min) was measured by spectrophotometry with an EnzytecTM kit (5380, ENZYTECTM) according to the manufacturer’s instructions. Amino acids were assayed with an amino acid analyzer (Biochrom 30, Biochrom). Molecules with a high molecular weight were precipitated by adding one volume of 25% (w/v) sulfosalicylic acid solution to 4 volumes of sample and then incubating for one hour at 4°C. After centrifugation (4°C, 10 min, 3°000 g), the sample was filtered through a 0.22 μm pore-sized nitrocellulose membrane filter (Millipore). Amino acids were separated by liquid chromatography on an ion-exchange column (Ultrapac-8 Lithium form; Amersham Pharmacia Biotech), and were detected by reaction with ninhydrin. The reaction was followed by absorbance measurement at 570 nm, except for proline, which was detected by absorbance at 440 nm. Norleucine (0.5 mM) was added to the samples as an internal standard.

Proteins were extracted from cells by incubation with 50% (v/v) DMSO for one hour at 105°C and were quantified with a BCA assay kit (BCA1, Sigma-Aldrich, France). The relative abundance of amino acids in biomass was assayed from cells hydrolysate (incubation 24 h at 110°C in presence of HCl 6 N) with the Biochrom analyser, as described below. The amount of nitrogen used for the synthesis of proteins was calculated from these data.

We used the copper permeabilization method to measure cytosolic and vacuolar amino acid pools [22]. Cells (3.10^8^) were harvested and washed three times with distilled water containing 150 mM of NaCl. Cells were then resuspended in 1.5 ml of 2.5 mM potassium phosphate buffer (pH 6.0) containing 0.6 M sorbitol, 10 mM glucose, and 0.2 mM CuCl_2_. After incubation for 10 min at 30°C, 1 ml of the cell suspension was filtered through a membrane filter (pore size, 0.45 μm; Sartorius) and was washed four times with 0.5 ml of the solution described above but without glucose or CuCl_2_. The filtrates were combined (3 ml) and were used as the cytosolic extract. The cells retained on the filter were resuspended in 3 ml of distilled water and boiled for 15 min. The suspension was centrifuged for 3 min at 5,000 rpm, and the supernatant was collected as the vacuolar extract.

### Estimation of dynamic variables

Cell growth was analyzed with a logistic model, which described the exponential growth phase followed by a stationary phase, as previously described by Crépin *et al.*, in 2012 [17]. The R software, version 2.15.3 was used for analysis [23].

In a similar approach, the dynamics of YAN, amino acid, and ammonium consumption during fermentation were fitted with a sigmoid or adapted Gompertz decay function, as described by Crépin *et al.*, in 2012 [17]. R software, version 2.15.3 was used for the analysis [23]. The equations obtained were then used to calculate the maximum rate of nitrogen (r_YANmax_), ammonium (r_NH4_) or amino acid (r_AA,_ for example, r_Arg_) consumption (Additional file 2: Figure S1).

### Statistical analyses

Statistical analyses were performed with R software, version 2.15.3 [23]. The phenotypic traits involving growth, fermentative capacity, and nitrogen consumption including DW, r_YAN_, T50, CO_2F_, r_CO2max_, and Y_DW/YAN_ were shown with bar plots. The effect of the initial nitrogen concentration (medium) on each variable of high (3 strains) and low (3 strains) biomass producers (group), was investigated by a two-way ANOVA. Fixed factors in the model were the group (high, low), the medium (SM45, SM85, SM165, SM260 and SM385) and their interaction. A *p*-value of 0.05 was considered statistically significant. In addition, the normality of residual distributions and homogeneity of variance were studied by standard diagnostic graphs; no violation of the assumptions was detected.

Groups were then compared for each medium. *P*-values of multiple comparisons were adjusted to a global threshold of 0.05 by the Hochberg FWER procedure to correct for multiplicity [24]. A multivariate factorial analysis (MFA) was performed to obtain an overview of the dataset, which consisted in 18 variables measured for six strains (3 high- and 3 low- biomass producing yeasts), grown on SM45, SM85, SM165, SM260 or SM385 medium [25]. The data set included a set of individuals described by three types of variables: the residual concentrations of Arg, Ala, Gln, NH_4_, Trp and Phe in the medium when 70% YAN had been consumed, the uptake rates of these nitrogen compounds evaluated by sigmoid models, and fermentative variables (DW, r_YAN_, T_50_, CO_2F_, r_CO2max_, and Y_DW/YAN_). The MFA takes into account the structure of the three groups of data and balances the influence of each group of variables. This enables the study of links between groups of variables to give an overview of the dataset.

One-way ANOVA was carried out to test a group effect (high- and low- biomass producers) on the following allocations: proteins, vacuole including vacuolar arginine, and cytoplasm. A *p*-value threshold of 0.05 without any adjustment for multiplicity was used and the strain was considered as random factor. For each variable, normality of residual distributions and homogeneity of variance were studied by standard diagnostic graphs; no violation of the assumptions was detected.

## Results

To better understand the basis of the high growth capacity of wine yeasts during fermentation, we further investigate the variability between strains in their ability to efficiently use nitrogen for growth. We selected three wine yeasts (L2226, WE372, and EC1118), referred as ‘high-biomass producers’, and three other *S. cerevisiae* strains displaying contrasting biomass yields from consumed nitrogen in presence of 165 mg.L^−1^ of YAN (YPS128, YPS1009, and NCYC110) [17]; these latter can be defined as ‘low-biomass producers’. All six strains were grown in duplicate in chemically defined media (SM45, SM85, SM165, SM260, SM385) containing 45, 85, 165, 260 or 385 mg.L^−1^ of YAN, respectively. YAN consisted of a mixture of NH_4_Cl and 18 amino acids providing 33% and 67% of YAN, respectively (Additional file 1: Table S1).

### YAN consumption, growth and fermentation performances of low and high biomass producers according to nitrogen availability

We first investigated the influence of the initial YAN content on the fermentative abilities of the two types of strains (low- and high- biomass producers). We examined six phenotypic traits (Table 2) related to growth and nitrogen consumption: (1) dry weight (DW); (2) time at which 50% of YAN was consumed (T_50_); (3) maximal rate of YAN consumption (r_YANmax_); (4) yield of biomass production with respect to consumed nitrogen (Y_DW/YAN_); and factors of fermentative performances, comprising (5) total amount of CO_2_ released (CO_2F_) and (6) maximal rate of CO_2_ production (r_CO2max_).

**Table 2:** **Fermentative capacity, growth and nitrogen consumption traits of six** ***S. cerevisiae*** **strains during fermentations containing various nitrogen concentrations**

**Media**	**Strains**	**DW**	**r**_**YANmax**_	**T**_**50**_	**CO**_**2F**_	**r**_**CO2max**_	**Y**_**DW/YAN**_
		**g.L** ^**−1**^	**mg N.L** ^**−1**^ **.h** ^**−1**^	**h**	**g.L** ^**−1**^	**g.L** ^**−1**^ **.h** ^**−1**^	**g.g** ^**−1**^
SM45	EC1118	0.9 ± 0.01	5.9 ± 0.86	14.1 ± 0.71	110 (na)	0.4 ± 0.03	20.7 ± 0.16
	L2226	nd	6.3 (na)	15.5 (na)	nd	0.5 ± 0.01	nd
	WE372	nd	4.8 ± 0.57	15.0 (na)	nd	0.4 ± 0.01	nd
	NCYC110	0.6 ± 0	4.8 ± 0.41	22.3 ± 0.32	90 (na)	0.5 ± 0.04	14.5 ± 0.01
	YPS1009	nd	3.9 ± 0.08	17.5 (na)	nd	0.4 ± 0.01	nd
	YPS128	0.9 ± 0.0	6.7 ± 0.33	18.0 (na)	102 (na)	0.4 ± 0.01	21.3 ± 0.32
SM85	EC1118	1.6 ± 0.02	10.6 ± 0.60	15.5 ± 0.14	111 (na)	0.9 ± 0.01	19.6 ± 0.25
	L2226	1.7 (na)	11.3 (na)	15.0 (na)	111 (na)	1.0 (na)	20.9 (na)
	WE372	1.9 (na)	11.6 (na)	nd	111 (na)	1.1 (na)	22.2 (na)
	NCYC110	1.1 (na)	8.6 (na)	22.7 (na)	86 (na)	0.9 (na)	13.5 (na)
	YPS1009	1.3 (na)	8.8 (na)	17.9 (na)	99 (na)	0.9 (na)	15.9 (na)
	YPS128	1.3 ± 0.02	9 ± 0.53	18.4 ± 0.42	104 (na)	0.8 ± 0.01	15.5 ± 0.30
SM165	EC1118	3.2 ± 0.04	24.3 ± 0.08	16.6 ± 0.14	112 ± 1.4	2.8 ± 0.08	19.4 ± 0.22
	L2226	3.2 ± 0.18	27.8 ± 0.44	15.3 ± 0.85	113 ± 0.1	2.5 ± 0.22	19.5 ± 1.12
	WE372	3.4 ± 0.15	24.4 ± 0.08	15.1 ± 0.07	115 ± 3.2	2.3 ± 0.07	21.1 ± 0.92
	NCYC110	2.1 ± 0.14	16.8 ± 0.79	22.6 ± 0.28	97 ± 2.6	2.1 ± 0.07	13.0 ± 0.87
	YPS1009	2.4 ± 0.14	21.1 ± 0.19	17.5 ± 0.28	99 ± 2.3	2.0 ± 0.04	14.7 ± 0.87
	YPS128	2.4 ± 0.18	21.1 ± 1.12	18.1 ± 0.14	105 ± 0.8	1.7 ± 0.07	14.7 ± 1.12
SM260	EC1118	3.4 ± 0.12	30.5 (na)	17.0 (na)	113 (na)	2.5 ± 0.28	13.2 ± 0.48
	L2226	3.5 (na)	31.6 (na)	15.5 (na)	112 (na)	2.5 (na)	13.5 (na)
	WE372	3.8 (na)	31.5 (na)	16.5 (na)	111 (na)	2.4 (na)	14.7 (na)
	NCYC110	2.8 (na)	20.8 (na)	21.2 (na)	101 (na)	2.4 (na)	10.8 (na)
	YPS1009	2.8 ± 0.09	25.1 ± 0.16	20.0 ± 0.14	102 ± 0.1	2.2 ± 0.23	10.9 ± 0.31
	YPS128	2.8 ± 0.12	24.9 (na)	18.0 (na)	105 (na)	2.1 ± 0.16	11.1 ± 0.48
SM385	EC1118	4.6 ± 0.08	30.2 ± 0.15	20.5 ± 0.21	113 ± 0.9	2.7 ± 0.04	14.4 ± 0.27
	L2226	5.3 ± 0.04	40.9 ± 0.03	18.7 (na)	110 ± 0.0	3.4 ± 0.01	14.6 ± 0.10
	WE372	5.1 ± 0.01	31.6 ± 0.01	20.5 (na)	110 ± 0.0	2.8 ± 0.01	15.8 ± 0.46
	NCYC110	3.9 ± 0.31	18.4 ± 0.67	25.5 (na)	111 ± 1.9	2.9 ± 0.07	12.7 ± 0.34
	YPS1009	3.4 ± 0.12	24.5 ± 0.02	22.5 (na)	108 ± 1.1	2.1 ± 0.01	13.3 ± 0.46
	YPS128	3.5 ± 0.11	20.6 ± 1.02	23 ± 0.42	105 ± 6.6	2.1 ± 0.03	12.7 ± 0.59
	Type	10.86***	8.44***	42.73***	53.91***	2.96***	32.57***
	Media	85***	82.77***	34**	12.65	91.86***	50.78***
	Type by Media	2.34**	4.69 **	0.43	9.71	1.65	5.77
	Residual	1.8	4.1	22.85	23.73	3.54	10.88
	Adj.R squared ^a^	97	94	66	65	95	83

DW: dry weight, r_Xmax_: maximal growth rate, T_50_: time at which 50% of YAN was consumed, r_YANmax_: maximal rate of YAN consumption, CO_2F_: total amount of CO_2_ released, r_CO2max_: maximal CO_2_ production rate, and Y_DW/YAN_: yield of biomass production with respect to consumed nitrogen. Mean values and standard errors were calculated from two replicates. Two-way ANOVA was carried out on each variable: Fixed factors were the type of strain (high or low), and the media (SM45, SM85, SM165, SM260, or SM385), and their interaction. A *p*-value threshold of 0.05 was considered significant.

na: not applicable (missing replicate); nd: not determined (not measured).

^a^Ajusted R square (%).

**, significant at p-value < 0.01, ***, significant at p-value < 0.001.

Analysis of variance showed a large diversity within the data set that was mostly explained (65 to 97% depending on the variable) by a combination of the composition of the medium and the strain (*p*-values < 0.01). However, these factors contributed differently to the variability of each phenotypic trait. The formation of biomass, the rate of CO_2_ production, and the rate of nitrogen consumption were substantially influenced by the nitrogen availability in the medium whereas T_50_, CO_2F_ and Y_DW/YAN_ were largely dependent on the type of strain (Table 2).

**Figure 1:**
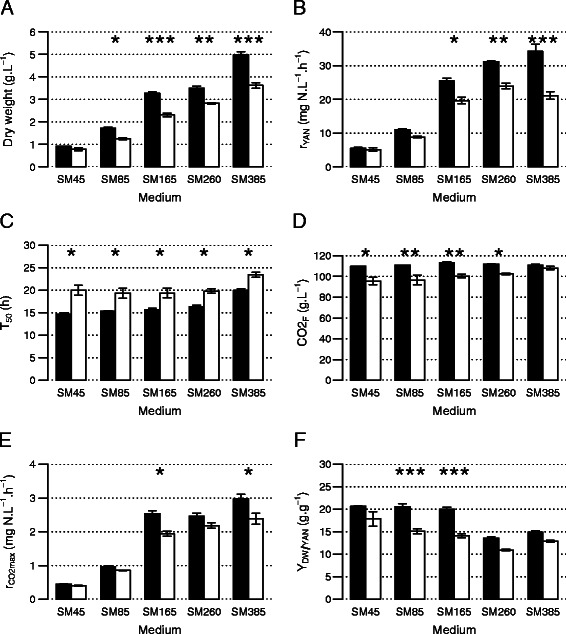
**Effect of initial nitrogen concentration on (A) dry weight, DW; (B) maximal rate of YAN consumption, r**_**YAN**_ **; (C) time required to consume 50% YAN, T**_**50**_ **; (D) final amount of CO**_**2**_ **released, CO**_**2F**_ **; (E) maximal rate of CO**_**2**_ **release, r**_**CO2max**_ **; and (F) yield of biomass production with respect to consumed YAN, Y**_**DW/YAN**_ **.** Low biomass-producing strains are shown by white bars and high biomass producing strains are shown by black bars. Mean values and standard error of the mean (SEM) were calculated from two replicates. The SEM is indicated by vertical error bars. The results of comparisons between the types of strain in each media are indicated inside the barplot^a^. Comparisons were corrected for multiplicity testing by the Hochberg method. ^a^: *, significant at *p*-value < 0.05, **, significant at *p*-value < 0.01.

**Figure 2:**
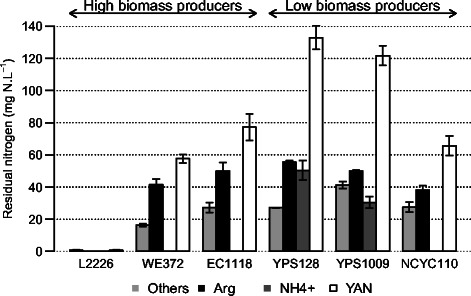
**Residual nitrogen sources (mg N.L** ^**−1**^ **) at the end of nitrogen consumption for cultures of six strains in medium SM385.** Other: sum of Met, Lys, Phe, Ile, Leu, His, Thr, Ser, Val, Tyr, Asp, Glu, Gln, Ala, Gly and Trp. Mean values and standard errors of the mean (SEM) were calculated from two replicates. SEM are indicated by vertical error bars.

Interestingly, we found that the initial partitioning between high and low biomass producers held true for all initial YAN concentrations tested, except for the lowest one (Figure 1). This relationship was not dependent on the phenotypic trait examined. Generally, high biomass producing strains displayed higher abilities to carry out alcoholic fermentation (r_CO2max_, CO_2F_) and to efficiently use nitrogen (r_YANmax_, T_50_, Y_DW/YAN_) than the low biomass producers. Nevertheless, under extreme conditions of nitrogen limitation in SM45 medium, the two types of strain could not be distinguished according to their growth or kinetic performances; however, under these conditions, only wine yeasts were able to complete 240 g.L^−1^ of glucose fermentation, resulting in a CO_2_ release of 110 g.L^−1^ (Additional file 1: Table S2). Both type of strain produced more biomass and had a higher rate of metabolic activity as nitrogen became increasingly available (Table 2, Figure 1). In contrast to other variables including DW, the variable Y_DW/YAN_ (biomass yield) decreased as the initial YAN concentration increased from 85 mg N.L^−1^ to 260 mg N.L^−1^ for all strains. However, under conditions of nitrogen excess (SM385), the biomass yield was slightly higher than that during fermentation in SM260. A substantial part of the available nitrogen was not consumed by either low- or high-biomass producers under conditions of nitrogen excess (SM385) (Figure 2), in contrast to media containing up to 260 mg N.L^−1^. For all strains, a large part of the residual nitrogen (between 55 and 128 mgN.L^−1^) was in the form of arginine, which is a poor nitrogen source.

### Profiles of consumption of nitrogen sources by low- and high-biomass producing yeasts

**Figure 3:**
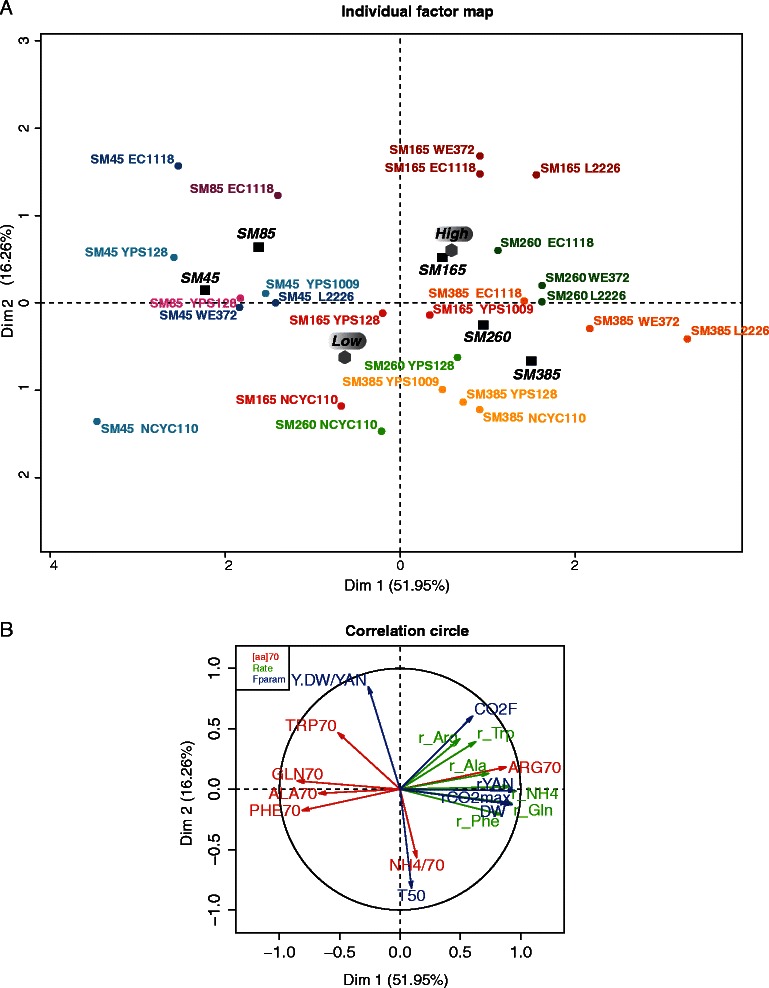
**Multiple Factors Analysis (MFA) of 18 variables measured for the six strains (3 high and 3 low biomass-producing strains) growing on SM45, SM85, SM165, SM260 and SM385 media. (A)** Map representing the individuals on the two first axes (explaining 68% of the total inertia). **(B)** Map representing the three types of variables on the two first axes: in red, the contribution of six nitrogen sources (selected as exhibiting the most variability) to residual nitrogen in the medium when 70% of YAN had been consumed; in green, the uptake rates of the nitrogen compounds evaluated by sigmoïd models; in blue, fermentative variables.

We aimed to investigate the origin of the differences in YAN consumption, biomass formation, and fermentative performance between high- and low-biomass producers. We examined the assimilation profiles of all nitrogen sources for the six strains growing on SM45, SM85, SM165, SM260, or SM385 (Additional file 2: Figures S2 and S3). We used these profiles to assess the contribution of the N-compounds to the residual nitrogen fraction in the medium after exhaustion of 70% of the N-resource. From the descriptive graphs, we selected six nitrogen sources: ammonium, glutamine, arginine, tryptophan, alanine and phenylalanine, based on their differential consumption by low- and high- biomass producers and on their relative abundance in the medium. We defined a data set involving the rate of consumption of these nitrogen sources and their contribution to residual nitrogen (r_GLN_, GLN_70_, r_NH4_, NH_4/70_, r_Arg_, ARG_70_, r_Ala_, ALA_70_, r_Trp_, TRP_70_, r_Phe_, PHE_70_), in addition to growth and fermentative traits previously described (Y_DW/YAN,_ DW, CO_2F_, r_CO2max_, r_YAN_, T_50_). These variables were measured during fermentations carried out with the six strains on the five media, and were subjected to an MFA analysis (Additional file 1: Table S2, Figure 3).

The first axis accounted for 52% of the total variation and was mostly defined by the significantly positively correlated variables r_CO2max_, r_YAN_, r_Gln_, r_NH4_, DW and, to a lesser extent, r_Ala_ and r_Phe_. The variables r_Ala_ and r_Phe_ were both negatively associated with the presence of glutamine, alanine, and phenylalanine in the medium after consumption of 70% YAN (GLN_70_, ALA_70_, PHE_70_). The biomass yield, Y_DW/YAN_, the rate of arginine and tryptophan consumption, (r_Arg,_ r_Trp_) and the amount of ammonium and tryptophan in the residual YAN fraction (NH_4/70_, TRP_70_,) were the variables that contributed most to the second principal component explaining more than 16% of the variance. The amount of arginine in the residual YAN fraction (ARG_70_) also moderately contributed to this second principal component. Strains were separated in the space defined by these two components according to the nitrogen concentration of the growth medium (from SM45 to SM385) (Figure 3). This separation was mostly along the first axis. This is consistent with an activation of yeast metabolism involving growth, substrate consumption, and fermentative activity in response to an increase in nitrogen availability.

Yeasts were also clearly distinguished into the two classes: high- and low-biomass producers. This distinction was observed for each medium, except for SM45, in which the nitrogen limitation was too stringent. However, the variables contributing to this distinction depended on the nitrogen availability in the medium (Figure 3, Additional file 1: Table S2). During fermentation in the presence of nitrogen excess (SM385) the two groups were separated mostly by the first component, including their rates of YAN, ammonium, and glutamine consumption, which resulted in a large amount of arginine in the residual nitrogen fraction. At lower nitrogen concentrations, the variables Y_DW/YAN_, CO_2F_, and the consumption rate of arginine and tryptophan, which are nitrogen sources consumed at the end of the growth phase, greatly contributed to the discrimination between wine commercial strains and other yeasts. The rates of YAN assimilation and ammonium consumption made small contributions to the distinction between yeasts at low nitrogen concentrations. Consequently, when the yeasts were cultivated on medium with a low nitrogen concentration (SM85), the two groups were distinguished almost exclusively by phenotypic traits contributing to the second axis. Under these conditions, the low biomass formation with respect to consumed nitrogen was mostly explained by a low rate of arginine and tryptophan consumption and by a large part of residual nitrogen as ammonium (NH_4/70_) after consumption of 70% YAN. Overall, the MFA highlighted the various factors in line with nitrogen assimilation that may be involved in the differences between strains in their ability to produce biomass. The contribution of each factor depended on the nitrogen availability in the medium.

### Comparison of nitrogen allocation between low- and high-biomass producers

**Figure 4:**
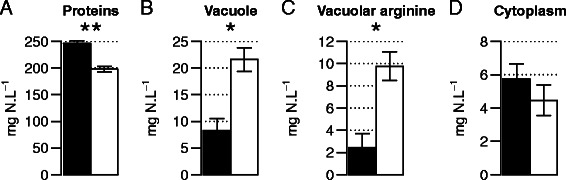
**Amount of nitrogen in high biomass producers (black) and low biomass producers (white) in SM260 at the beginning of the stationary phase; (A) in the form of proteins; (B) in the vacuole; (C) in the form of arginine in the vacuole; and (D) in the cytoplasm.** Mean values and standard errors of the mean (SEM) were calculated from two or three replicates. SEM are indicated by vertical error bars. The results of one way ANOVA are indicated inside the barplot^a^. ^a^: *, significant at p-value < 0.05, **, significant at p-value < 0.01.

Under conditions of nitrogen limitation, biomass yield from consumed nitrogen and the rate of arginine uptake made a large contribution to the distinction between high and low biomass-producing strains. This suggests that the two types of yeast allocate nitrogen sources differently. We compared the proportions of nitrogen incorporated into proteins, stored in vacuoles, or found in the cytosol between high (WE372 and EC1118) and low (YPS128 and YPS1009) biomass-producers. This analysis was carried out with yeast at the end of the growth phase in SM260 fermentations (Table 3, Figure 4).

**Table 3:** **Growth, nitrogen consumption, and nitrogen fate for four strains at the end of nitrogen consumption and for two strains at the point of 50% nitrogen consumption in SM260**

		**50% YAN consumption**		**End of YAN consumption**			
		**EC1118**	**YPS128**	**WE372**	**EC1118**	**YPS128**	**YPS1009**
**Population**	10^6^ cell.mL^−1^	45.5 ± 1.2	51.8 ± 1.7	135.2 ± 0.1	129.8 ± 1.0	109.2 ± 3.1	112.0 ± 1.2
**Vol**	μm^3^	73.2 ± 0.5	63.3 ± 1.0	56.3 ± 0.5	61.6 ± 0.2	74.4 ± 1.0	67.4 ± 1.4
**DW**	g.L^−1^	1.55 ± 0.02	1.44 ± 0.02	3.20 ± 0.02	3.20 ± 0.03	2.70 ± 0.01	2.70 ± 0.12
**YANc**							
**Total**	mgN.L^−1^	160 ± 1	143 ± 1	258 ± 1	258 ± 1	258 ± 1	258 ± 1
**NH4+**	mgN.L^−1^	60.4 ± 0.3	46.9 ± 0.7	61.5 ± 0.4	61.5 ± 0.4	61.5 ± 0.4	61.5 ± 0.4
**Arg**	mgN.L^−1^	20.4 ± 0.3	20.0 ± 0.4	78.5 ± 0.5	78.5 ± 0.5	78.5 ± 0.5	78.5 ± 0.5
**Proteins**							
	% (g.g^−1^)	56.9 ± 0.3	56.5 ± 0.4	47.6 ± 0.4	47.3 ± 0.2	46.0 ± 0.1	44.8 ± 0.7
	mgN.L^−1^	142 ± 2	130 ± 1	247 ± 0	245 ± 2	202 ± 2	192 ± 0
**Vacuole**							
**Total**	mgN.L^−1^	9.5 ± 0.1	6.3 ± 0.3	6.7 ± 0.2	9.4 ± 0.2	20.2 ± 0.7	23.7 ± 0.3
**Arg**	mgN.L^−1^	3.7 ± 0.2	4.1 ± 0.1	1.7 ± 0.0	2.9 ± 0.1	10.7 ± 0.3	8.4 ± 0.2
**Cytoplasm**							
**Total**	mgN.L^−1^	4.7 ± 0.0	3.9 ± 0.1	6.4 ± 0.2	5.3 ± 0.1	5.0 ± 0.1	3.6 ± 0.1

PopM: maximal cellular population (10^6^ cell .mL^−1^), Vol: cellular volume (μm^3^), DW: dry weight (g.L^−1^), YANc: yeast assimilable nitrogen consumed (mg N.L^−1^), Y_DW/YAN_: yield of biomass production from nitrogen consumed (g.g^−1^), Proteins: percentage of proteins (g.L^−1^) divided by the dry weight (g.L^−1^) (%) or amount of nitrogen found in proteins (mg N.L^−1^), Vacuole: amount of nitrogen in the vacuole, Total (mg N.L^−1^) and as Arginine (mg N.L^−1^), and Cytoplasm: amount of nitrogen found in the cytoplasm (mg N.L^−1^).

Mean values and standard errors were calculated from three replicates (except for strains WE372 and YPS1009 for which only duplicates were used).

We first confirmed that the strains exhibited substantial differences in the final amount of biomass produced (at least 20% more for high biomass-producers), but not in the amount of nitrogen consumed (260 mg N.L^−1^) (Table 3). Proteins accounted for a similar fraction of dry weight (45 to 48%) for all the yeasts (Table 3). Consequently, the total amount of nitrogen allocated to proteins was substantially and significantly (*p*-value < 0.01) lower for low biomass-producers, (202 mg N.L^−1^ for YPS128 and 192 mg N.L^−1^ for YPS1009), than for high biomass-producers, (245 mg N.L^−1^ for EC1118 and 247 mg N.L^−1^ for WE372). More than 94% of nitrogen consumed was used for proteins synthesis in the high biomass-producers.

The cytoplasmic content of free amino acids did not differ between strains, and represented only a small proportion of all assimilated nitrogen (around 5 mg N.L^−1^). In contrast, the fraction of nitrogen accumulated in vacuoles differed substantially between strains. There was three times more nitrogen in the vacuolar compartment of low biomass producers (20.2 mg N.L^−1^ for YPS128 and 23.7 mg N.L^−1^ for YPS1009) than in high biomass producers (6.7 mg N.L^−1^ for WE372 and 9.4 mg N.L^−1^ for EC1118). Analysis of the amino acids composition of vacuolar fractions revealed that arginine was the most abundant amino acid stored in vacuoles. There was 2.5 times more arginine in the vacuolar compartment of low biomass-producing strains (10.7 mgN.L^−1^for YPS128 and 8.4 mgN.L^−1^ for YPS1009) than in that of high biomass-producers (1.7 mgN.L^−1^for WE372 and 2.9 mgN.L^−1^ for EC1118) (*p*-value < 0.05 Figure 4). We also investigated the intracellular fate of nitrogen during the growth phase of EC1118 (high biomass producer) and YPS128 (low biomass producer). Surprisingly, no difference was found between strains in the allocation of nitrogen (Table 3) at this stage of culture. Indeed, most assimilated nitrogen was directed towards protein synthesis (88 to 91%), whereas only a small amount was stored in vacuoles (4.4 to 5.9%) or was present in the free pool of amino acids (2.7 to 2.9%). Arginine accounted for a larger percentage of the vacuolar pool of amino acids in YPS128 (65%) than in EC1118 (39.5%). The intracellular storage of arginine in EC1118 was lower in the stationary phase than in the growth phase, whereas the amount of vacuolar arginine in YPS128 was higher at the end of the growth (10.7 mg N.L^−1^) than during the growth phase (4.1 mg N.L^−1^). This suggests that the use of arginine (including vacuolar arginine) for biosynthesis during the late stages of growth is more efficient for high biomass producers than for low biomass producers.

**Figure 5:**
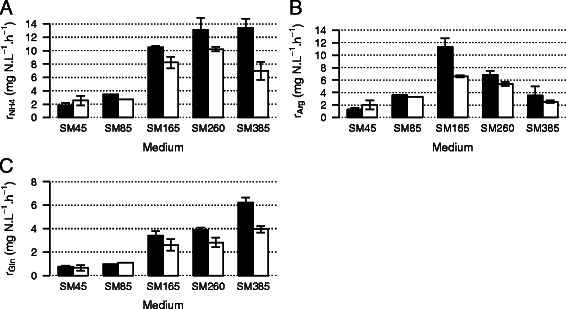
**Effects of the initial concentration of nitrogen on the uptake rate of (A) ammonium r**_**NH4+**_ **(mg N.L** ^**−1**^ **.h** ^**−1**^ **); (B) arginine r**_**Arg**_ **(mg N.L** ^**−1**^ **.h** ^**−1**^ **); and (C) glutamine r**_**Gln**_ **(mg N.L** ^**−1**^ **.h** ^**−1**^ **).** Low biomass-producing strains are shown by white bares and high biomass-producing strains are shown by black bars. Mean values and standard error of the mean (SEM) were calculated from two replicates. The SEM is indicated by vertical error bars.

We found only small differences in the rate of arginine assimilation between the classes of yeast, with the exception of yeast grown on SM165 (Figure 5). This was likely due to the extreme conditions of nitrogen limitation on SM45 and SM85, and to the preferential use of other nitrogen sources on SM260 and SM385. However, in contrast to the uptake of other nitrogen compounds, the rate of arginine uptake was low in yeasts grown on SM385, and was half the value of that during fermentation on SM260. This was coherent with the presence of residual arginine under nitrogen excess conditions, reflecting a preferential use by yeasts of favored nitrogen sources that more efficiently promote their growth.

We examined the consumption of the most abundant preferred nitrogen sources. We found large variations between high and low biomass producers in their rate of uptake of ammonium, and to a lesser extent, glutamine (Figure 5). The size of these differences was positively correlated with the nitrogen availability in the medium. During fermentations on SM385, the rate of consumption of glutamine for low biomass producers was 3.9 mg N.L^−1^.h^−1^ and that of ammonium was 7.0 mg N.L^−1^.h^−1^. However, under these conditions, the rate of consumption of glutamine for high biomass producing strains was 6.2 mg N.L^−1^.h^−1^ and that of ammonium was 13.5 mg N.L^−1^.h^−1^. Consequently, the low biomass-producers did not completely consume the available ammonium ions under these conditions; YPS128 only consumed 65% and YPS1009 consumed 77%. This explains why the YAN consumption of these strains was lower than that of other strains (Figure 2). Thus, low biomass producers have a limited ability to take up ammonium and glutamine in the presence of nitrogen excess, which may contribute to their low rate of YAN assimilation and results in only the partial consumption of ammonium.

## Discussion

During industrial alcoholic fermentations, and particularly during winemaking, the formation of biomass plays a key role both in driving the fermentation rate and in controlling metabolites formation [8,9,26]. *S. cerevisiae* species shows significant diversity in biomass yield from nitrogen, and consequently in the fermentative capacity [15,16]. In particular, wine yeasts appeared as high biomass producing strains, able to complete the fermentation of large sugar concentrations. We report here that the profile of YAN consumption, and particularly the rate of YAN uptake, is highly variable and depends on both the strain and on nitrogen availability. We previously observed a significant correlation between YAN consumption, the final production of biomass, and the fermentative capacity, during fermentations of 14 *S. cerevisiae* strains under N-limiting conditions [17]. We now extend this correlation to a range of nitrogen concentrations, from limitation to excess. The capacity of yeasts to efficiently consume nitrogen resources appeared to be a key factor affecting their growth, even if the other factors related to wine environment (as osmotic or acid stresses) can also contribute to differences between strains.

Little was known about the origin of strain-specific differences in the use of N-compounds for biomass production and its dependence on nitrogen availability. A comparative analysis of the fermentative behavior of nine food-processing strains in three media representative of baking, brewing and wine-making revealed that the environment is the main factor shaping alcoholic fermentation, followed by genetic factors [9]. Consistent with this finding, we varied the YAN availability in the medium to show that the environment has a large and significant effect on the fermentative behavior of yeast strains. Our results confirm both that culture conditions have a major effect on strain performance and that initial nitrogen concentration is an important factor for the course of wine fermentation. Interestingly, when nitrogen is plentiful, yeast preferentially use particular nitrogen compounds to optimize their growth (glutamine and ammonium at the expense of the poor nitrogen source: arginine). This allows yeasts to adjust their growth capacity according to the availability of nitrogen sources.

Furthermore, we found that strains clustered into two groups, according to their ability to produce biomass and their capacity to consume nitrogen. This clustering was irrespective of the initial nitrogen availability, supporting the contribution of genetic factors to phenotypic variability. High biomass producers were able to complete the fermentation of 240 g.L^−1^ glucose even in highly nitrogen-deficient medium (not shown), and consumed nitrogen more quickly than the other yeasts. Of note, these high biomass producers originate from industrial wine processes. Thus, the highly efficient use of limiting nitrogen resources may be a trait that contributes to the adaptation of wine yeasts to the stresses of their particular environment.

Several mechanisms may contribute to the variability in the capacity of strains to efficiently use available nitrogen. The active uptake of amino acids and ammonium is dependent on the proton gradient across the plasma membrane that is driven by the H^+^-ATPase Pma1p [27–29]. Modulation of ATPase activity results in substantial changes in the transport kinetics of all the nitrogen compounds across the plasma membrane [30]. Consequently, strain-specific differences in the amount of biomass produced from nitrogen may be explained by differences in the rate of YAN uptake amongst strains, involving variations in ATPase activity, or more generally, in other global regulatory mechanisms that control the entry of all nitrogen sources to the cell. However, we report that nitrogen uptake rate depends on the particular transported nitrogen compound, which is inconsistent with a strong influence of mechanisms regulating global transport. An alternative mechanism may be differences in the rate of consumption of various nitrogen sources, in particular the most abundant ones. Consistent with this, high biomass producing strains exhibited higher uptake rates of ammonium, and to a lesser extent, higher uptake of glutamine than low biomass producing yeasts. During wine fermentation, the ammonium concentration does not exceed 10 mM and the uptake of this nitrogen source is entirely mediated by three permeases in *S. cerevisiae*: Mep1p, Mep2p, and Mep3p [31–34]. These transporters display different kinetic properties and are differentially regulated [32,35,36]. Several factors may be involved in the highly efficient uptake of ammonium by high biomass producers under conditions of nitrogen excess; MEP genes may be highly expressed and these strains may have highly efficient ammonium transporters. A comparative genomic analysis of *MEP1*, *MEP2*, and *MEP3* sequences between the 14 strains (data not shown) failed to identify SNPs in the coding sequences that may explain any potential differences in the activity of these transporters in high biomass-producing strains. The *MEP* genes are tightly regulated, mostly by nitrogen catabolic repression (NCR). Their transcription is activated by the binding of the activating transcription factors Gln3p and Gat1p to GATA promoter sequences [18,37]. If the concentration of ammonium is low or if non-preferred nitrogen sources are the only source available, these regulatory proteins are translocated to the nucleus resulting in the up-regulation of the transcription of *MEP* genes. These regulatory proteins return to the cytoplasm when preferred nitrogen sources become available [31,32]. Genetic background affects the efficiency of NCR control [38]. Therefore, the difference in the rate of ammonium consumption between high and low biomass-producers may be due to differences in NCR regulation of *MEP* genes.

Strain-specific differences in the use of N-compounds for biomass formation may also be related to differences in the intracellular fate of consumed YAN. We report large differences in the fraction of nitrogen allocated to protein synthesis between high and low biomass-producing strains, mostly associated with differences in the efficiency of arginine catabolism. Arginine is one of the most abundant nitrogen sources in grape juice and constitutes around 25% of YAN [12]. Nevertheless, arginine is considered to be a non-preferred nitrogen source, because it supports poorly yeast growth [39], and is the most stored amino acid in the vacuole during the growth phase [20]. We quantitatively analyzed the fate of consumed YAN. After complete exhaustion of nitrogen sources at the end of the growth phase, the amount of amino acids stored in the vacuole, especially arginine, was higher for low biomass producers than for high biomass producers. These differences were less apparent during the growth phase, in which the two groups of strains accumulated a similar amount of amino acids in the vacuole. These data indicate that high biomass producers store poor nitrogen sources during the first stages of culture and, instead, use rich nitrogen sources that promote the efficient production of biomass. Then, as nitrogen becomes limiting, these strains metabolize the stored nitrogen for further growth. This is consistent with previous work reporting that the total amino acid content of vacuoles increases gradually up to the middle of the logarithmic phase, and decreases thereafter, as the cells reach the stationary phase [20]. By contrast, low biomass producers were unable to effectively re-mobilize and use nitrogen accumulated in the vacuole (mostly consisting of arginine) at the end of the growth phase. This may explain the lower proportion of nitrogen allocated to protein synthesis in these strains than in high biomass producers.

## Conclusion

In summary, we demonstrate that the high ability of some *S. cerevisiae* strains to produce biomass during wine fermentation is related to efficient management of the available nitrogen resources under conditions of either nitrogen excess or limitation. The rate of ammonium uptake by high biomass producers was higher than that of low biomass producers, resulting in a high rate of consumption of this rich nitrogen source when nitrogen was over supplied. Another characteristic of high biomass-producing strains was their enhanced capacity to use amino acids previously stored in the vacuole, particularly arginine, during the late stages of growth. This enabled protein synthesis when nitrogen became limiting. Interestingly, high biomass producers are commercial wine strains, and the yeast strains from other industrial processes or natural environments gave low yields of biomass from consumed nitrogen. The efficient consumption of nitrogen sources of wine strains under nitrogen limiting conditions may likely contribute to their adaptation to the environment.

## Additional files

Additional file 1: Table S1. Initial concentration of ammonium ions and amino acids in the media used in this study. **Table S2.** Phenotypic variables used for MFA. Contribution of alanine, arginine, glutamine, phenylalanine, tryptophan and ammonium to residual nitrogen. Uptake rate of the most abundant nitrogen compounds. Fermentative variables. Additional file 2: Figure S1. Examples of dynamics of YAN, amino acids and ammonium consumption (mgN.L-1) during fermentation fitted using a sigmoid or adapted Gompertz decay function. **Figure S2.** Contribution of ammonium and amino acids to residual nitrogen in the medium after exhaustion of 70% of the N ressource. Data are expressed in %. SM45: grey, SM85: black, SM165: white, SM260: medium grey and SM385: dark grey. **Figure S3.** Kinetics of consumption of the nitrogen sources during fermentation of low - (YPS1009, YPS128, NCYC110) and high- (WE372, EC1118, L2226) biomass producers on SM45, SM85, SM165, SM260 and SM385 media.
