# Supplementary material for: Efficient ammonium uptake and mobilization of vacuolar arginine by Saccharomyces cerevisiae wine strains during wine fermentation
Source: Microb Cell Fact. 2014 Aug 19;13:109. doi: 10.1186/s12934-014-0109-0 (PMC4244049; doi:10.1186/s12934-014-0109-0)
Supplement: Additional file 1: Table S1. — Initial concentration of ammonium ions and amino acids in the media used in this study. Table S2. Phenotypic variables used for MFA. Contribution of alanine, arginine, glutamine, phenylalanine, tryptophan and ammonium to residual nitrogen. Uptake rate of the most abundant nitrogen compounds. Fermentative variables. [file 12934_2014_109_MOESM1_ESM.docx]

**Table S1**: Initial concentration of ammonium ions and amino acids in the media used in this study

| **Nitrogen sources** | **SM45**  **mg.L^-1^** | **SM85**  **mg.L^-1^** | **SM165**  **mg.L^-1^** | **SM260**  **mg.L^-1^** | **SM385**  **mg.L^-1^** |
| --- | --- | --- | --- | --- | --- |
| Lys | 0.15 | 0.31 | 0.61 | 0.92 | 1.53 |
| Cys | 1.53 | 3.05 | 6.10 | 9.15 | 15.25 |
| Gly | 2.14 | 4.27 | 8.54 | 12.81 | 21.35 |
| Tyr | 2.14 | 4.27 | 8.54 | 12.81 | 21.35 |
| Met | 3.66 | 7.32 | 14.64 | 21.96 | 36.60 |
| Ile | 3.81 | 7.63 | 15.25 | 22.88 | 38.13 |
| His | 3.81 | 7.63 | 15.25 | 22.88 | 38.13 |
| Phe | 4.42 | 8.85 | 17.69 | 26.54 | 44.23 |
| Asp | 5.19 | 10.37 | 20.74 | 31.11 | 51.85 |
| Val | 5.19 | 10.37 | 20.74 | 31.11 | 51.85 |
| Leu | 5.64 | 11.29 | 22.57 | 33.86 | 56.43 |
| Thr | 8.85 | 17.69 | 35.38 | 53.07 | 88.45 |
| Ser | 9.15 | 18.30 | 36.60 | 54.90 | 91.50 |
| Glu | 14.03 | 28.06 | 56.12 | 84.18 | 140.30 |
| Ala | 16.93 | 33.86 | 67.71 | 101.57 | 169.28 |
| Trp | 20.89 | 41.79 | 83.57 | 125.36 | 208.93 |
| Arg | 43.62 | 87.23 | 174.46 | 261.69 | 436.15 |
| NH4Cl | 53.50 | 107.00 | 214.00 | 321.00 | 535.00 |
| Gln | 58.87 | 117.73 | 235.46 | 353.19 | 588.65 |
| Pro* | 71.37 | 142.74 | 285.48 | 428.22 | 713.70 |

SM45: synthetic medium with 45 mg N.L^-1^ of YAN; SM85: synthetic medium with 85 mg N.L^-1^ of YAN; SM165: synthetic medium with 165 mg N.L^-1^ of YAN; SM260: synthetic medium with 260 mg N.L^-1^ of YAN; SM385: synthetic medium with 385 mg N.L^-1^ of YAN.

*In the absence of oxygen, proline is not assimilable by yeast, and therefore was not considered in calculations of assimilable nitrogen.

**Table S2**: Phenotypic variables used for MFA.

***Contribution of alanine, arginine, glutamine, phenylalanine, tryptophan and ammonium to residual nitrogen.***

| **Medium** | **Strain** | **Type** | **ALA_70_** | **ARG_70_** | **GLN_70_** | **NH_4/70_** | **PHE_70_** | **TRP_70_** |
| --- | --- | --- | --- | --- | --- | --- | --- | --- |
| SM45 | EC1118 | High | 13.69 | 21.56 | 18.70 | 11.34 | 0.43 | 17.45 |
| SM45 | L2226 | High | 9.79 | 21.67 | 14.48 | 25.84 | 0.83 | 10.76 |
| SM45 | WE372 | High | 8.93 | 19.05 | 11.50 | 29.98 | 0.68 | 12.69 |
| SM45 | NCYC110 | Low | 16.36 | 0.00 | 17.78 | 33.60 | 1.32 | 16.44 |
| SM45 | YPS1009 | Low | 9.69 | 13.10 | 12.16 | 31.44 | 0.00 | 17.12 |
| SM45 | YPS128 | Low | 10.83 | 8.52 | 12.77 | 32.15 | 1.02 | 17.87 |
| SM85 | EC1118 | High | 10.53 | 27.13 | 7.31 | 19.14 | 0.40 | 17.24 |
| SM85 | YPS128 | Low | 9.89 | 23.73 | 4.45 | 25.60 | 0.91 | 18.46 |
| SM165 | EC1118 | High | 11.54 | 46.28 | 6.67 | 14.52 | 0.09 | 13.84 |
| SM165 | L2226 | High | 7.90 | 46.44 | 1.10 | 25.69 | 0.00 | 14.45 |
| SM165 | WE372 | High | 7.78 | 39.15 | 3.49 | 26.92 | 0.22 | 17.18 |
| SM165 | NCYC110 | Low | 14.98 | 21.55 | 6.30 | 36.19 | 0.53 | 13.90 |
| SM165 | YPS1009 | Low | 11.90 | 37.62 | 5.70 | 22.12 | 0.70 | 13.34 |
| SM165 | YPS128 | Low | 6.13 | 37.92 | 2.03 | 33.34 | 0.52 | 14.60 |
| SM260 | EC1118 | High | 10.60 | 39.49 | 6.16 | 20.81 | 0.06 | 14.63 |
| SM260 | L2226 | High | 5.77 | 37.31 | 2.22 | 38.94 | 0.05 | 10.78 |
| SM260 | WE372 | High | 6.55 | 35.01 | 1.93 | 38.66 | 0.09 | 14.08 |
| SM260 | NCYC110 | Low | 12.70 | 18.19 | 6.20 | 42.36 | 0.32 | 13.37 |
| SM260 | YPS128 | Low | 4.01 | 33.92 | 1.40 | 40.37 | 0.40 | 15.41 |
| SM385 | EC1118 | High | 9.67 | 51.40 | 4.68 | 11.20 | 0.00 | 14.61 |
| SM385 | L2226 | High | 4.25 | 44.57 | 0.70 | 37.13 | 0.00 | 10.15 |
| SM385 | WE372 | High | 4.00 | 45.83 | 1.01 | 31.14 | 0.00 | 14.40 |
| SM385 | NCYC110 | Low | 10.92 | 37.13 | 4.37 | 29.72 | 0.05 | 11.57 |
| SM385 | YPS1009 | Low | 9.77 | 41.44 | 8.03 | 22.50 | 0.24 | 8.86 |
| SM385 | YPS128 | Low | 4.35 | 45.77 | 3.03 | 29.46 | 0.15 | 10.82 |

The contribution of amino acids to the residual nitrogen fraction was assessed as the concentrations of Arg, Ala, Gln, NH_4_, Trp and Phe in the medium when 70% YAN had been consumed.

***Uptake rate of the most abundant nitrogen compounds***

| **Medium** | **Strain** | **Type** | **r_Arg_** | **r_Ala_** | **r_Gln_** | **r_NH4_** | **r_Phe_** | **r_Trp_** |
| --- | --- | --- | --- | --- | --- | --- | --- | --- |
| SM45 | EC1118 | High | 1.82 | 0.58 | 0.85 | 2.50 | 0.04 | 0.81 |
| SM45 | L2226 | High | 1.18 | NA | 0.82 | 1.36 | NA | NA |
| SM45 | WE372 | High | 0.98 | 0.56 | 0.61 | 1.55 | 0.03 | 0.42 |
| SM45 | NCYC110 | Low | 1.85 | 0.94 | 0.61 | 1.83 | 0.05 | 0.67 |
| SM45 | YPS1009 | Low | 0.82 | 0.11 | 0.27 | 3.92 | NA | NA |
| SM45 | YPS128 | Low | 3.36 | 0.62 | 1.06 | 1.86 | 0.05 | 1.79 |
| SM85 | EC1118 | High | 3.67 | 1.02 | 0.98 | 3.46 | 0.10 | 1.25 |
| SM85 | YPS128 | Low | 3.32 | 0.93 | 1.07 | 2.75 | 0.08 | 0.63 |
| SM165 | EC1118 | High | 13.35 | 4.10 | 2.74 | 10.25 | 0.21 | 1.84 |
| SM165 | L2226 | High | 11.98 | 4.13 | 4.12 | 10.90 | 0.29 | 2.59 |
| SM165 | WE372 | High | 8.59 | 3.47 | 3.35 | 10.42 | 0.23 | 2.75 |
| SM165 | NCYC110 | Low | 6.38 | 3.77 | 1.71 | 6.56 | 0.20 | 1.23 |
| SM165 | YPS1009 | Low | 6.91 | 3.26 | 2.65 | 8.76 | 0.21 | 1.33 |
| SM165 | YPS128 | Low | 6.53 | 3.01 | 3.42 | 9.38 | 0.24 | 1.47 |
| SM260 | EC1118 | High | 5.68 | 2.48 | 3.48 | 12.29 | 0.27 | 3.40 |
| SM260 | L2226 | High | 7.73 | 3.12 | 3.87 | 10.65 | 0.32 | 2.42 |
| SM260 | WE372 | High | 7.14 | 3.52 | 4.23 | 16.49 | 0.29 | 2.46 |
| SM260 | NCYC110 | Low | 5.10 | 1.64 | 2.40 | 9.90 | 0.24 | 1.07 |
| SM260 | YPS128 | Low | 5.69 | 2.53 | 3.22 | 10.57 | 0.22 | 1.62 |
| SM385 | EC1118 | High | 1.41 | 1.55 | 5.48 | 11.55 | 0.51 | 1.00 |
| SM385 | L2226 | High | 6.31 | 4.15 | 6.85 | 16.06 | 0.74 | 3.20 |
| SM385 | WE372 | High | 2.83 | 2.88 | 6.38 | 12.70 | 0.55 | 1.81 |
| SM385 | NCYC110 | Low | 2.83 | 1.22 | 3.66 | 9.29 | 0.43 | 1.30 |
| SM385 | YPS1009 | Low | 2.59 | NA | 4.48 | 7.14 | 0.21 | 0.61 |
| SM385 | YPS128 | Low | 2.14 | 2.00 | 3.69 | 4.54 | 0.32 | 0.93 |

The uptake rates were evaluated by sigmoid models, as described in the Materials and Methods section.

***Fermentative variables***

| **Medium** | **Strain** | **Type** | **DW** | **r_YAN_** | **T_50_** | **CO_2F_** | **r_CO2max_** | **Y_DW/YAN_** |
| --- | --- | --- | --- | --- | --- | --- | --- | --- |
| SM45 | EC1118 | High | 0.92 | 5.94 | 14.10 | 109.78 | 0.44 | 20.72 |
| SM45 | L2226 | High | NA | 6.27 | 15.50 | NA | 0.48 | NA |
| SM45 | WE372 | High | NA | 4.84 | 15.00 | NA | 0.43 | NA |
| SM45 | NCYC110 | Low | 0.64 | 4.82 | 22.28 | 89.70 | 0.46 | 14.49 |
| SM45 | YPS1009 | Low | NA | 3.91 | 17.50 | NA | 0.38 | NA |
| SM45 | YPS128 | Low | 0.94 | 6.74 | 18.00 | 101.56 | 0.38 | 21.28 |
| SM85 | EC1118 | High | 1.64 | 10.58 | 15.50 | 110.82 | 0.90 | 19.58 |
| SM85 | YPS128 | Low | 1.30 | 9.04 | 18.40 | 103.90 | 0.84 | 15.54 |
| SM165 | EC1118 | High | 3.17 | 24.30 | 16.60 | 112.14 | 2.78 | 19.37 |
| SM165 | L2226 | High | 3.19 | 27.77 | 15.30 | 113.53 | 2.49 | 19.52 |
| SM165 | WE372 | High | 3.45 | 24.42 | 15.05 | 114.65 | 2.35 | 21.08 |
| SM165 | NCYC110 | Low | 2.12 | 16.78 | 22.60 | 97.20 | 2.06 | 12.97 |
| SM165 | YPS1009 | Low | 2.41 | 21.13 | 17.50 | 99.41 | 2.04 | 14.75 |
| SM165 | YPS128 | Low | 2.40 | 21.15 | 18.10 | 104.79 | 1.71 | 14.68 |
| SM260 | EC1118 | High | 3.41 | 30.49 | 17.00 | 112.65 | 2.47 | 13.17 |
| SM260 | L2226 | High | 3.50 | 31.58 | 15.50 | 111.70 | 2.53 | 13.53 |
| SM260 | WE372 | High | 3.80 | 31.50 | 16.50 | 110.98 | 2.37 | 14.69 |
| SM260 | NCYC110 | Low | 2.80 | 20.78 | 21.20 | 100.65 | 2.43 | 10.83 |
| SM260 | YPS128 | Low | 2.85 | 24.90 | 18.00 | 105.02 | 2.06 | 11.06 |
| SM385 | EC1118 | High | 4.56 | 30.21 | 20.45 | 112.98 | 2.73 | 14.37 |
| SM385 | L2226 | High | 5.33 | 40.92 | 18.70 | 110.00 | 3.38 | 14.63 |
| SM385 | WE372 | High | 5.07 | 31.58 | 20.50 | 110.00 | 2.85 | 15.80 |
| SM385 | NCYC110 | Low | 3.94 | 18.37 | 25.50 | 111.37 | 2.90 | 12.69 |
| SM385 | YPS1009 | Low | 3.45 | 24.51 | 22.50 | 107.60 | 2.15 | 13.32 |
| SM385 | YPS128 | Low | 3.49 | 20.58 | 23.00 | 105.20 | 2.13 | 12.67 |

DW: dry weight; r_YANmax_: maximal rate of YAN consumption; T_50_: time at which 50% of YAN was consumed; CO_2F_: total amount of CO_2_ released; r_CO2max_: maximal rate of CO_2_ production; Y_DW/YAN_: yield of biomass production with respect to consumed nitrogen.
