# Supplementary material for: Efficient ammonium uptake and mobilization of vacuolar arginine by Saccharomyces cerevisiae wine strains during wine fermentation
Source: Microb Cell Fact. 2014 Aug 19;13:109. doi: 10.1186/s12934-014-0109-0 (PMC4244049; doi:10.1186/s12934-014-0109-0)
Supplement: Additional file 2: Figure S1. — Examples of dynamics of YAN, amino acids and ammonium consumption (mgN.L-1) during fermentation fitted using a sigmoid or adapted Gompertz decay function. Figure S2. Contribution of ammonium and amino acids to residual nitrogen in the medium after exhaustion of 70% of the N ressource. Data are expressed in %. SM45: grey, SM85: black, SM165: white, SM260: medium grey and SM385: dark grey. Figure S3. Kinetics of consumption of the nitrogen sources during fermentation of low - (YPS1009, YPS128, NCYC110) and high- (WE372, EC1118, L2226) biomass producers on SM45, SM85, SM165, SM260 and SM385 media. [file 12934_2014_109_MOESM2_ESM.pdf]

Figure S1 : Examples of dynamics of YAN, amino acids and ammonium consumption (mg N.L^{-1}) during fermentation fitted using a sigmoid or adapted Gompertz decay function.

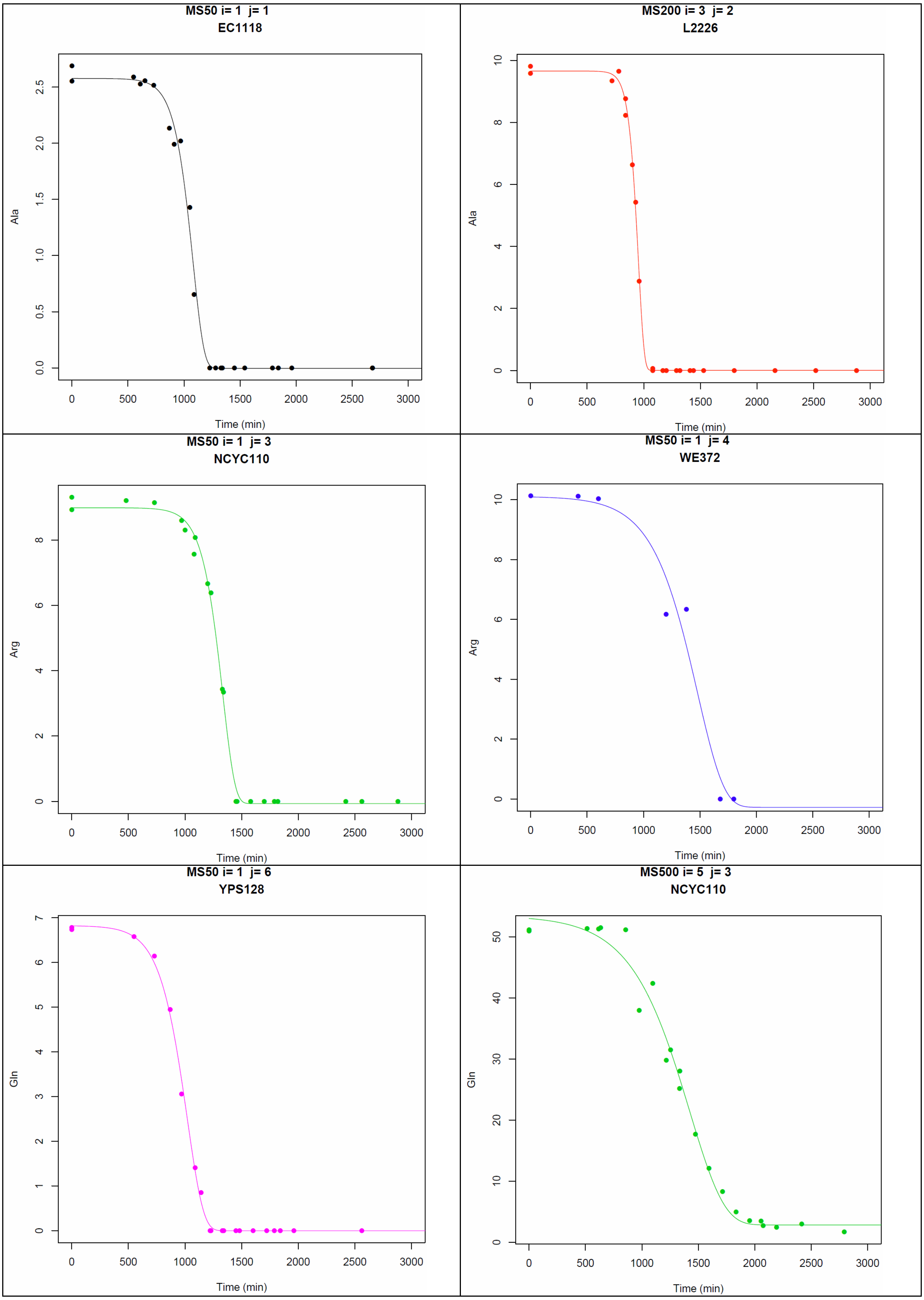

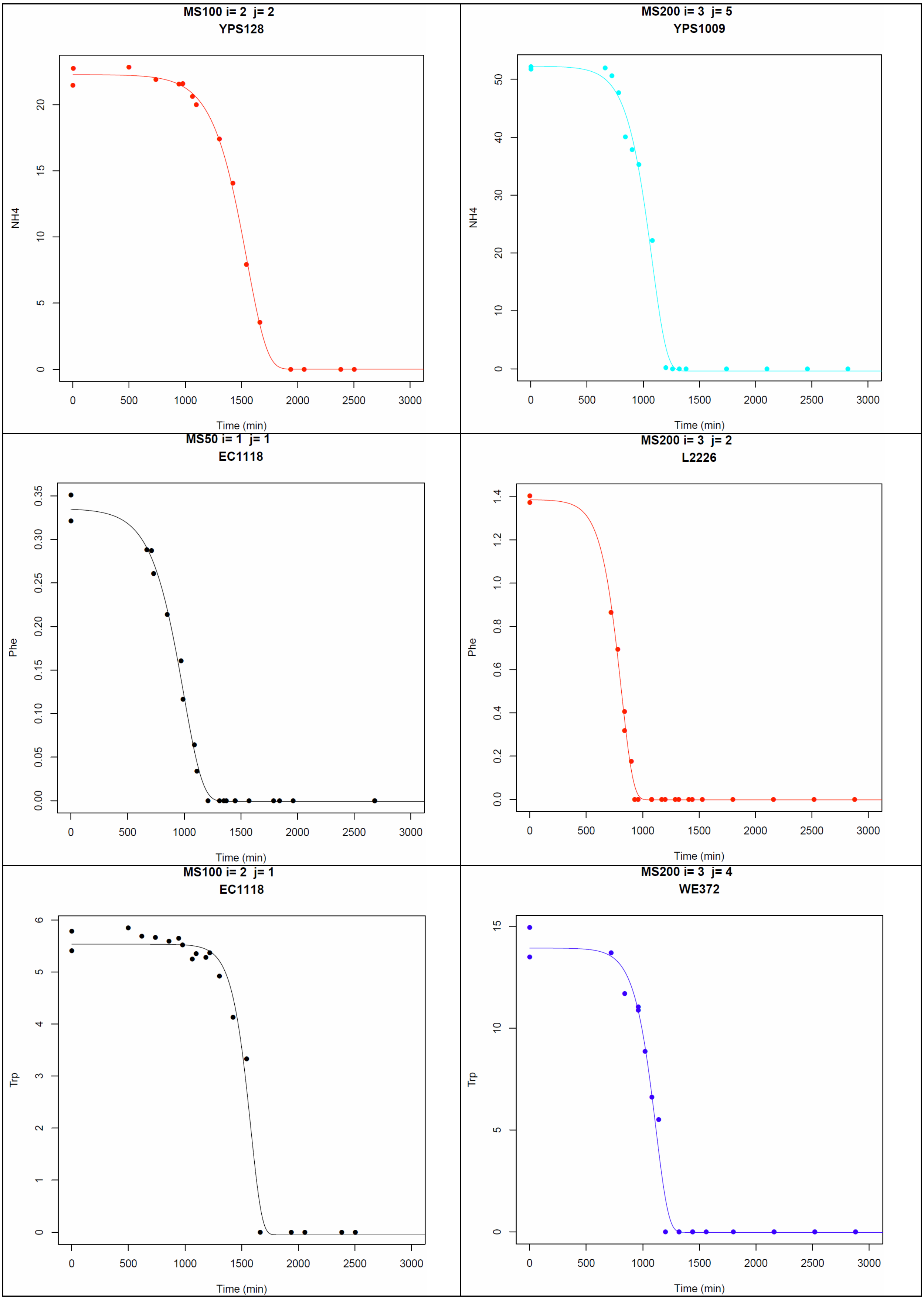
MS100 i= 2 j= 2
YPS128
NH4
Time (min)
MS200 i= 3 j= 5
YPS1009
NH4
Time (min)
MS50 i= 1 j= 1
EC1118
Phe
Time (min)
MS200 i= 3 j= 2
L2226
Phe
Time (min)
MS100 i= 2 j= 1
EC1118
Trp
Time (min)
MS200 i= 3 j= 4
WE372
Trp
Time (min)
0
500
1000
1500
2000
2500
3000

The maximal rates of consumption were estimated as follows :

Code snippet

(…)
```
x<-mydata_milieu_souche2[,"Temps"]
y<-mydata_milieu_souche2[,myparam]

yfin_0 = (min(y))
Delta_0 = max(y) - min(y)
M_0 = x[abs(y - max(y)/2) == min(abs(y - max(y)/2))]
K_0 = 0.5/60
res = try(nls(y~yfin + Delta*exp(-exp(K*(x-M))),
start=list(yfin=yfin_0,Delta = Delta_0,M=M_0,K=K_0),trace = F))

if(inherits(res,"try-error")){
    # If error, it just represents the points and is incremented
   plot(x,y,main=unique(mydata_milieu$Souches)[j],pch=16,col=j,
    xlim=c(0,6500),ylab=myparam)
  title(main=paste(unique(mydata$Milieu)[i],"i=",i," j=",j,sep=" "),
    outer=TRUE, line=-1)
next
} else {
  # else, results
  #---parameters storing
  yfin_f =  summary(res)$parameters[1,1]
  Delta_f = summary(res)$parameters[2,1]
  M_f = summary(res)$parameters[3,1]
  K_f = summary(res)$parameters[4,1]

  #--- simulation at all time points
  y_sim = yfin_f + Delta_f*exp(-exp(K_f*(x_sim-M_f)))
  #--- calculate the derivative of rate calculation for consumption,
  # delta between t and t+1
  deltas_conc = deltas_conc_spe = NULL
  for(l in 2:length(y_sim)){
      deltas_conc = c(deltas_conc,abs(y_sim[l] - y_sim[(l-1)]))
  }
  Rmax = max(abs(deltas_conc)) #  min
  Rmax60=Rmax*60               #  hour
(…)
}
(…)
```

Figure S2. Contribution of ammonium and amino acids to residual nitrogen in the medium after exhaustion of 70% of the N ressource. Data are expressed in %.
SM45: grey, SM85: black, SM165: white, SM260: medium grey and SM385: dark grey

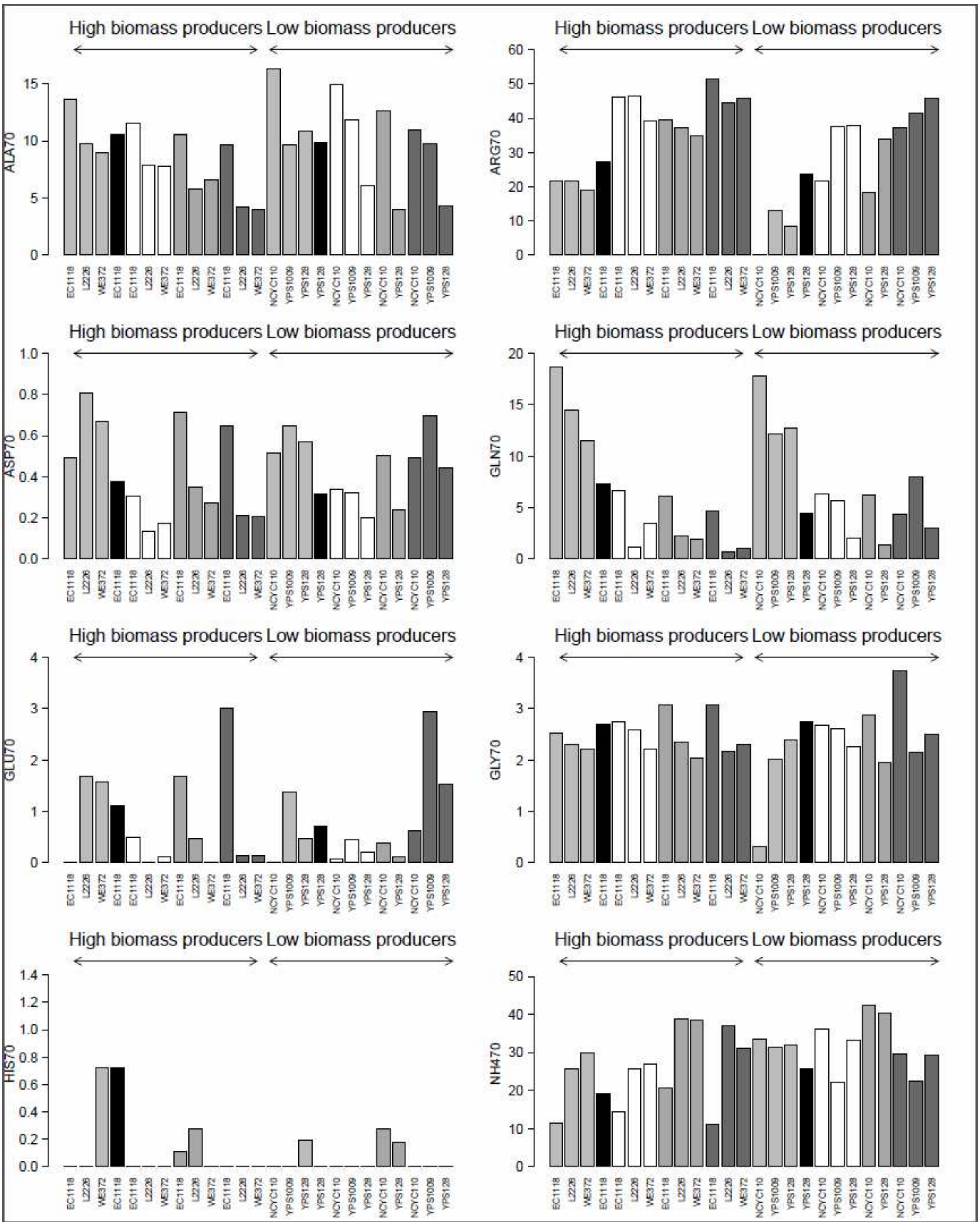

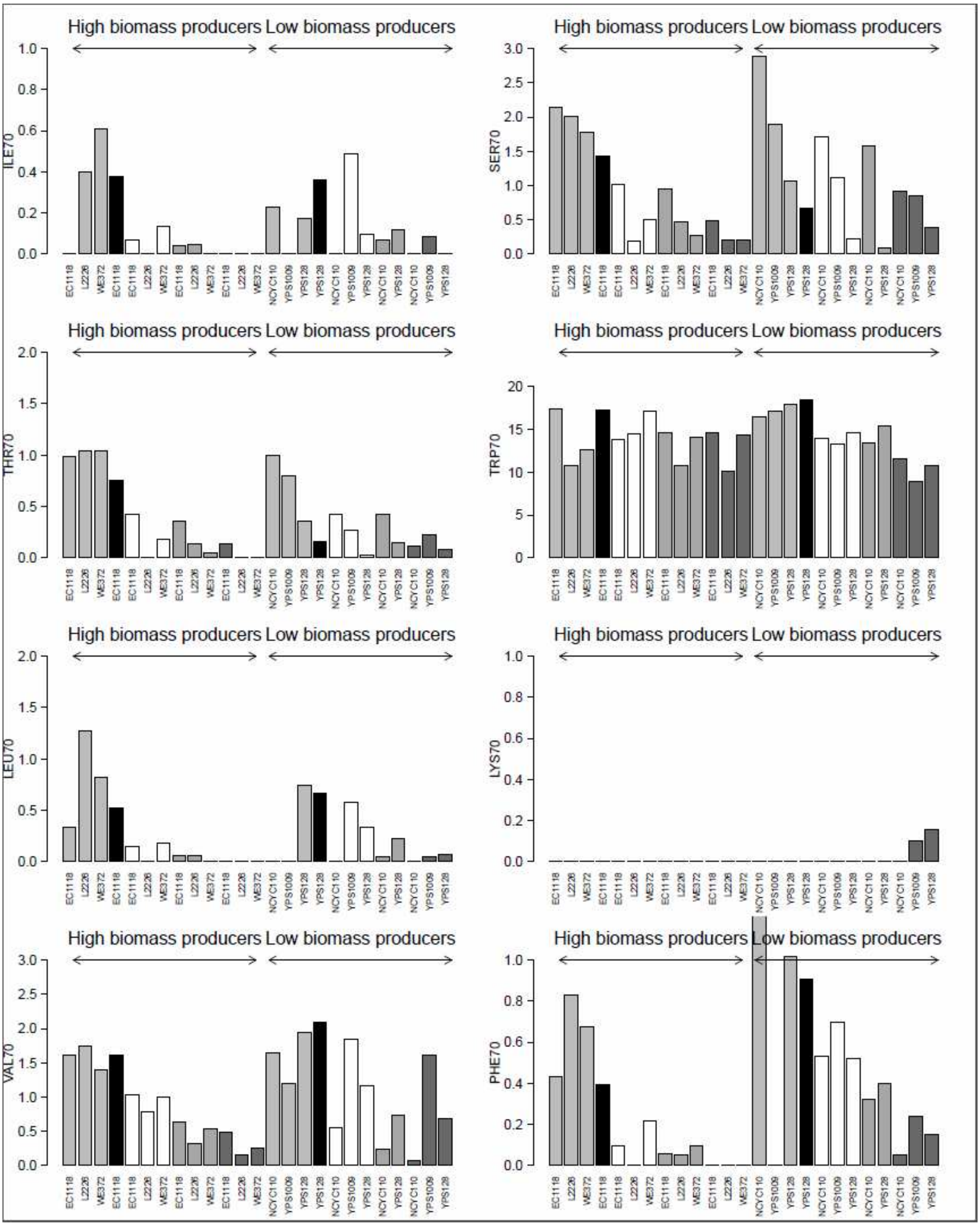
High biomass producers
Low biomass producers
ILE70
SER70
THR70
TRP70
LEU70
LYS70
VAL70
PHE70
EC1118 L2226 WE372 EC1118 L2226 WE372 EC1118 L2226 WE372 EC1118 L2226 WE372
NCYC110 YPS1009 YPS128 YPS128 NCYC110 YPS1009 YPS128 NCYC110 YPS128 NCYC110 YPS1009 YPS128

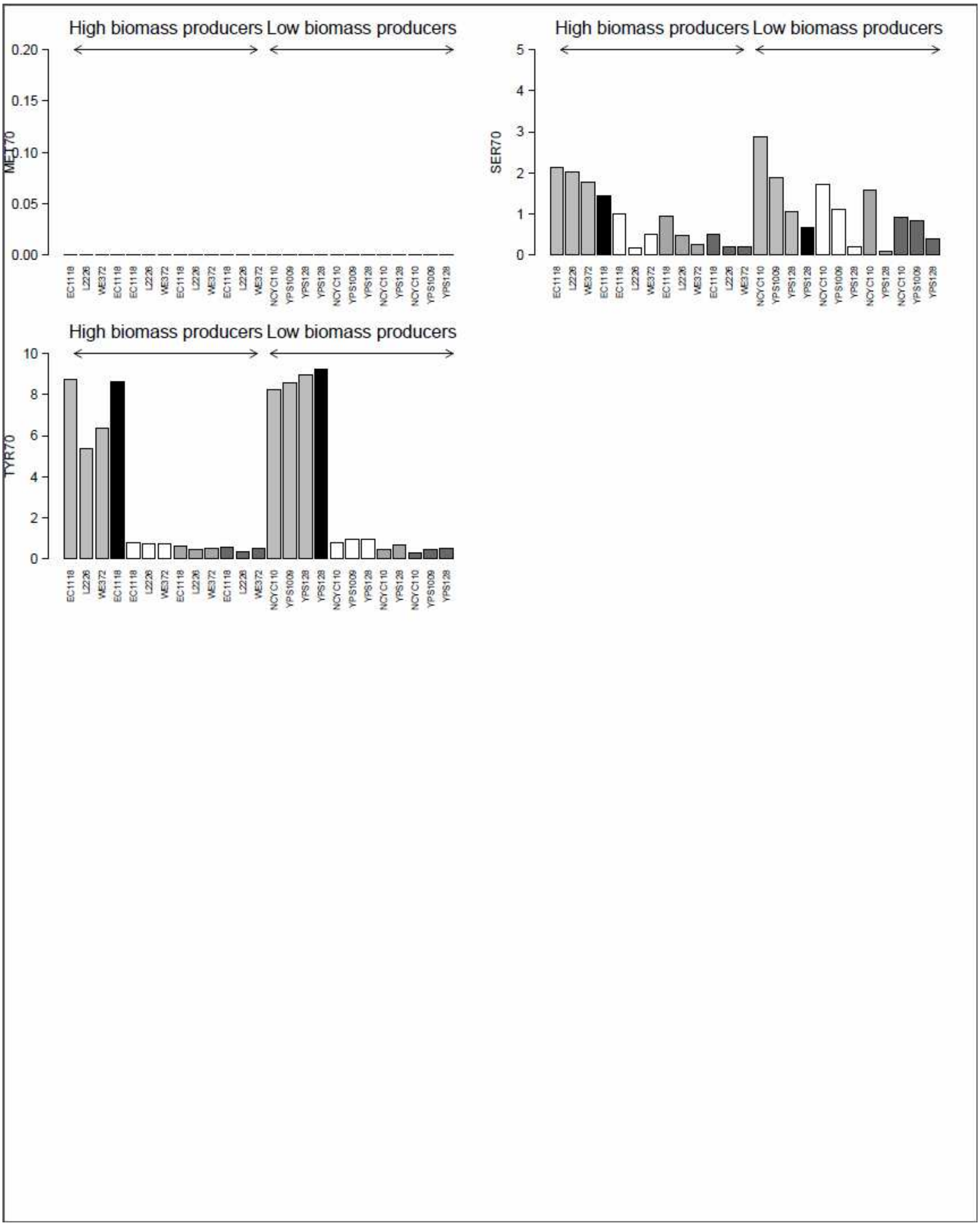

High biomass producers
Low biomass producers
MET70
0.00
0.05
0.10
0.15
0.20
SER70
0
1
2
3
4
5
TYR70
0
2
4
6
8
10
EC1118
L2226
WE372
NCYC110
YPS1009
YPS128

Figure S3. Kinetics of consumption of the nitrogen sources during fermentation of low- (YPS1009, YPS128, NCYC110) and high- (WE372, EC1118, L2226) biomass producers on SM45, SM85, SM165, SM260 and SM385 media.

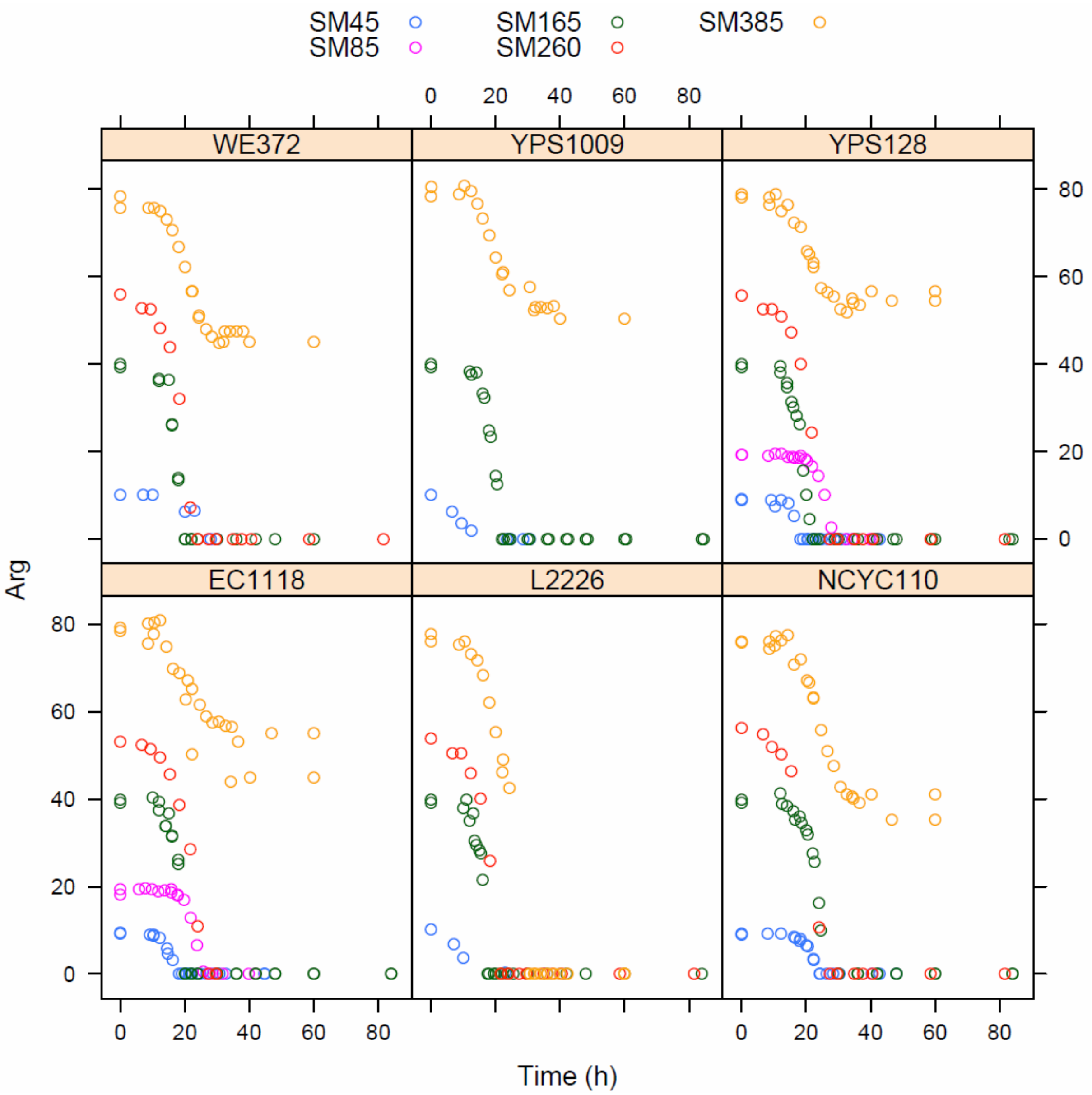

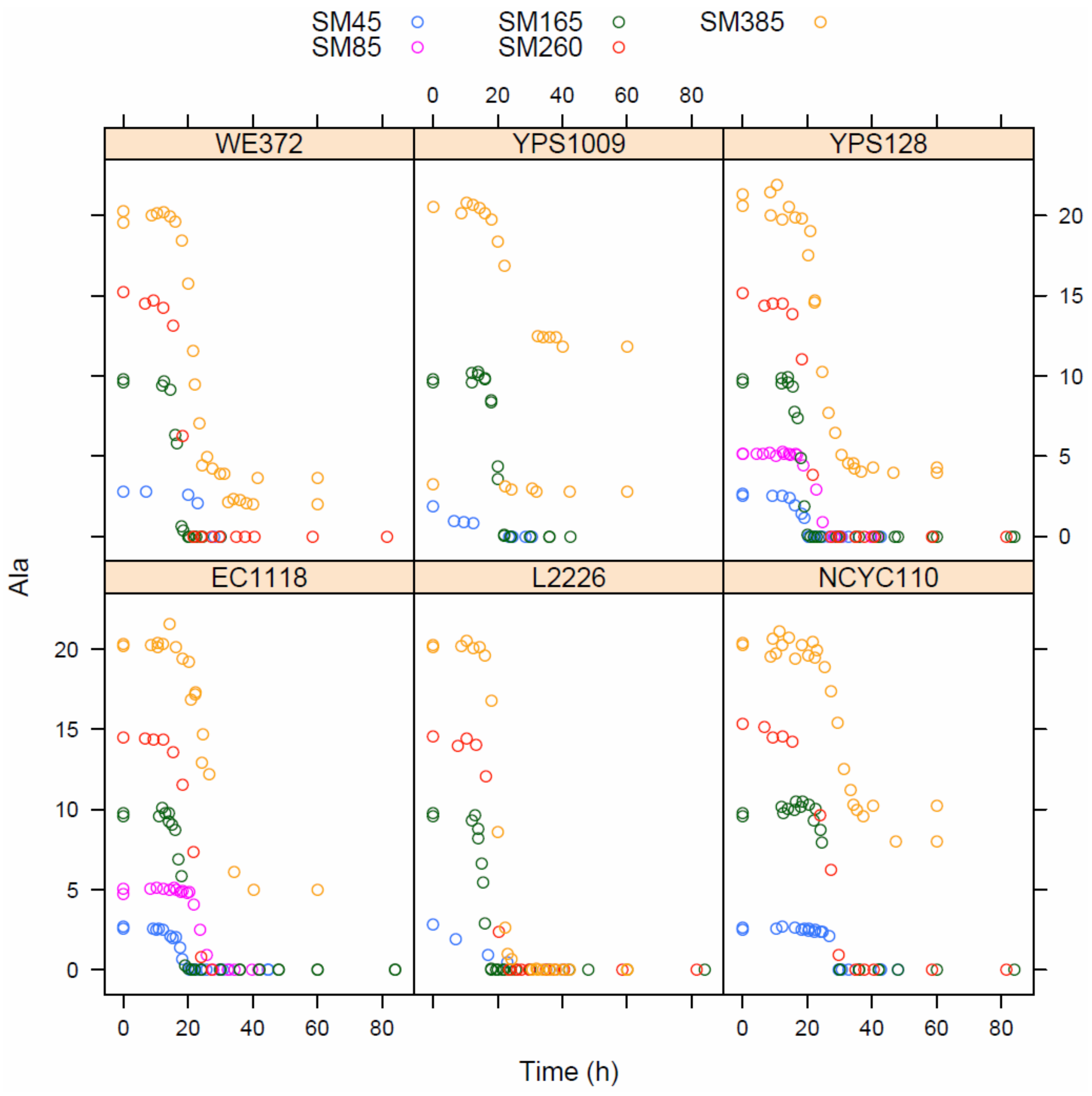
SM45
SM85
SM165
SM260
SM385
WE372
YPS1009
YPS128
EC1118
L2226
NCYC110
Ala
Time (h)
0
20
40
60
80
0
5
10
15
20

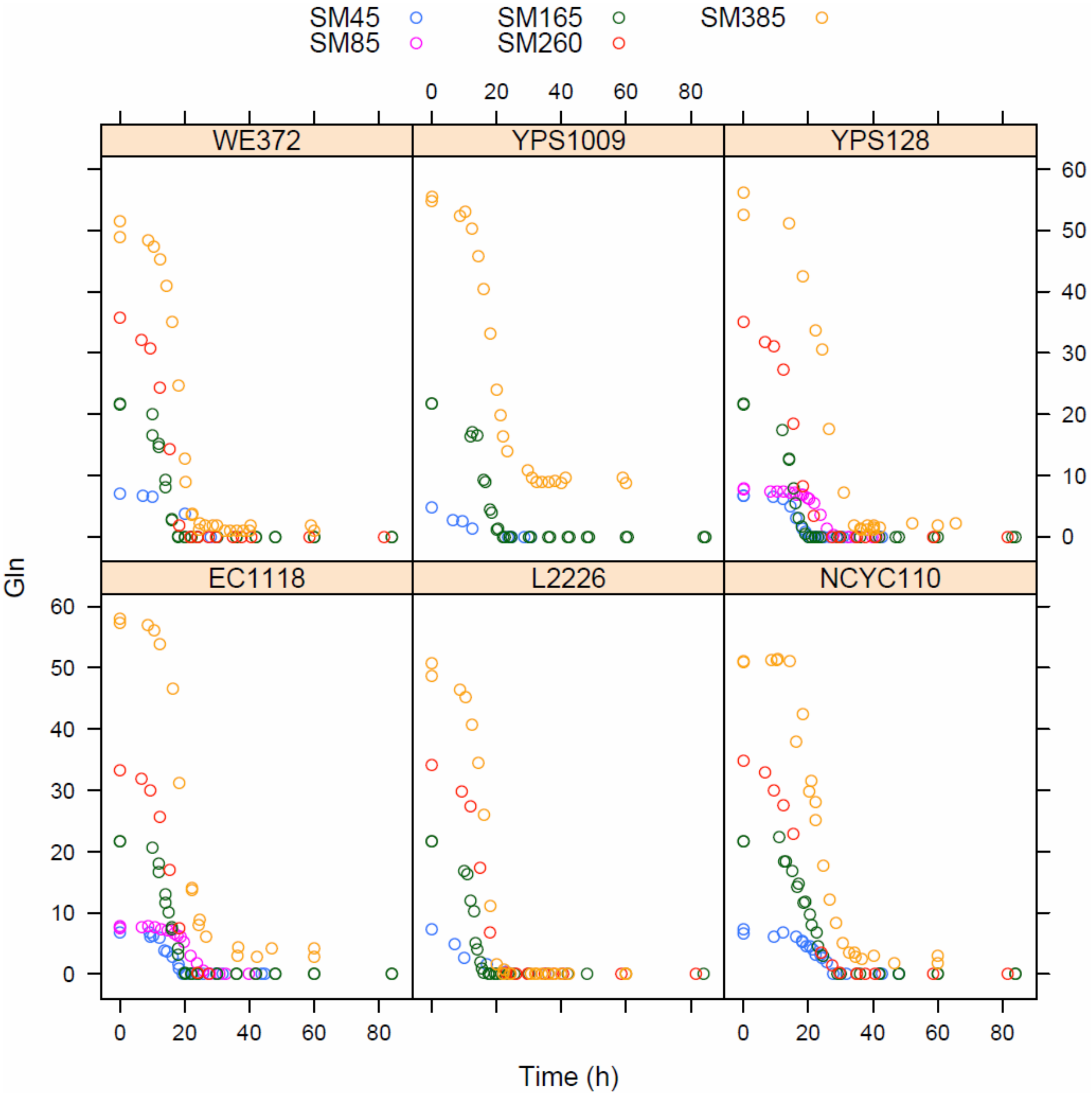
SM45
SM85
SM165
SM260
SM385
WE372
YPS1009
YPS128
EC1118
L2226
NCYC110
Gln
Time (h)
0
10
20
30
40
50
60
80

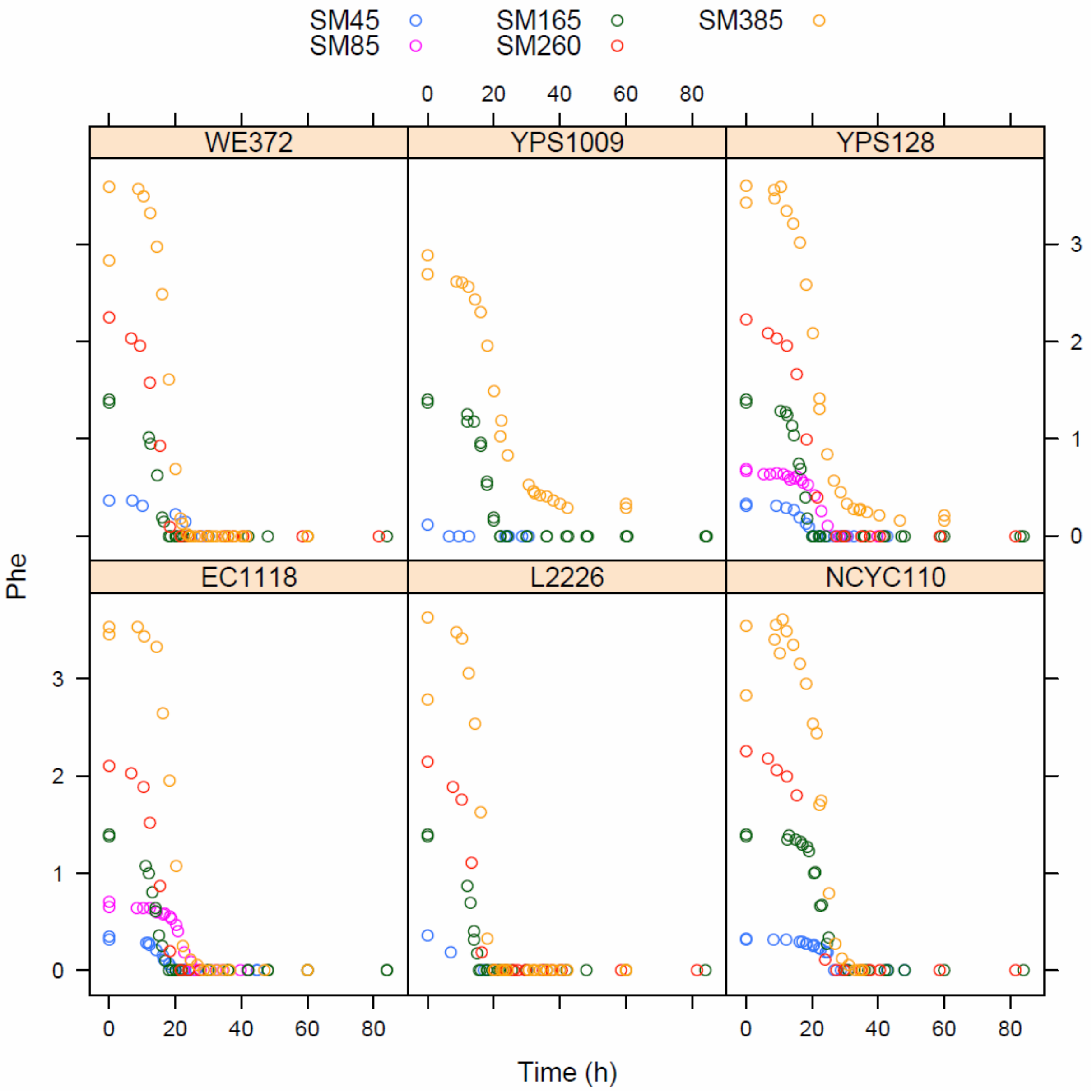

SM45
SM85
SM165
SM260
SM385
WE372
YPS1009
YPS128
EC1118
L2226
NCYC110
Phe
Time (h)
0
20
40
60
80
0
1
2
3

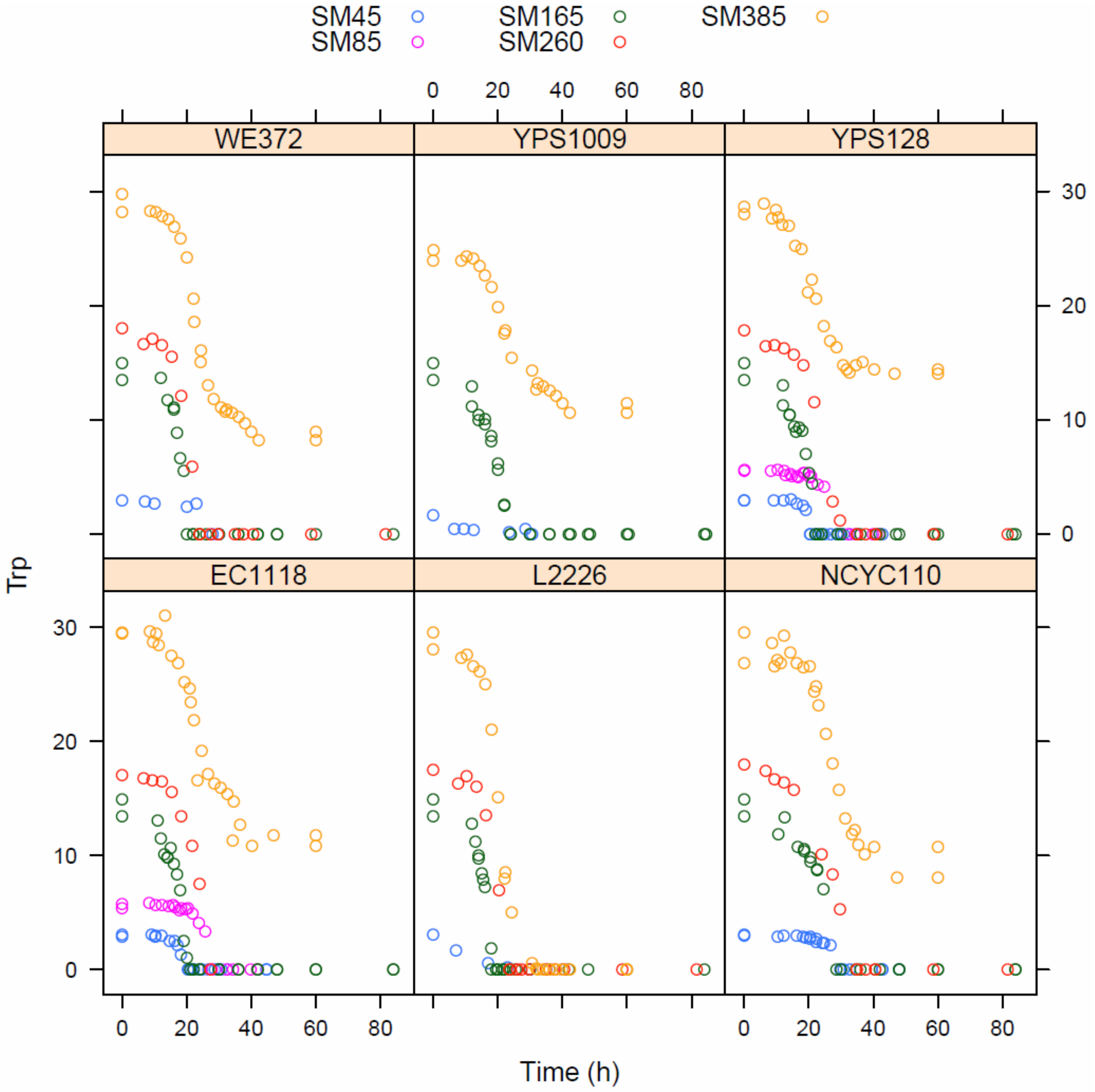
SM45
SM85
SM165
SM260
SM385
WE372
YPS1009
YPS128
EC1118
L2226
NCYC110
Trp
Time (h)
0
10
20
30
40
60
80

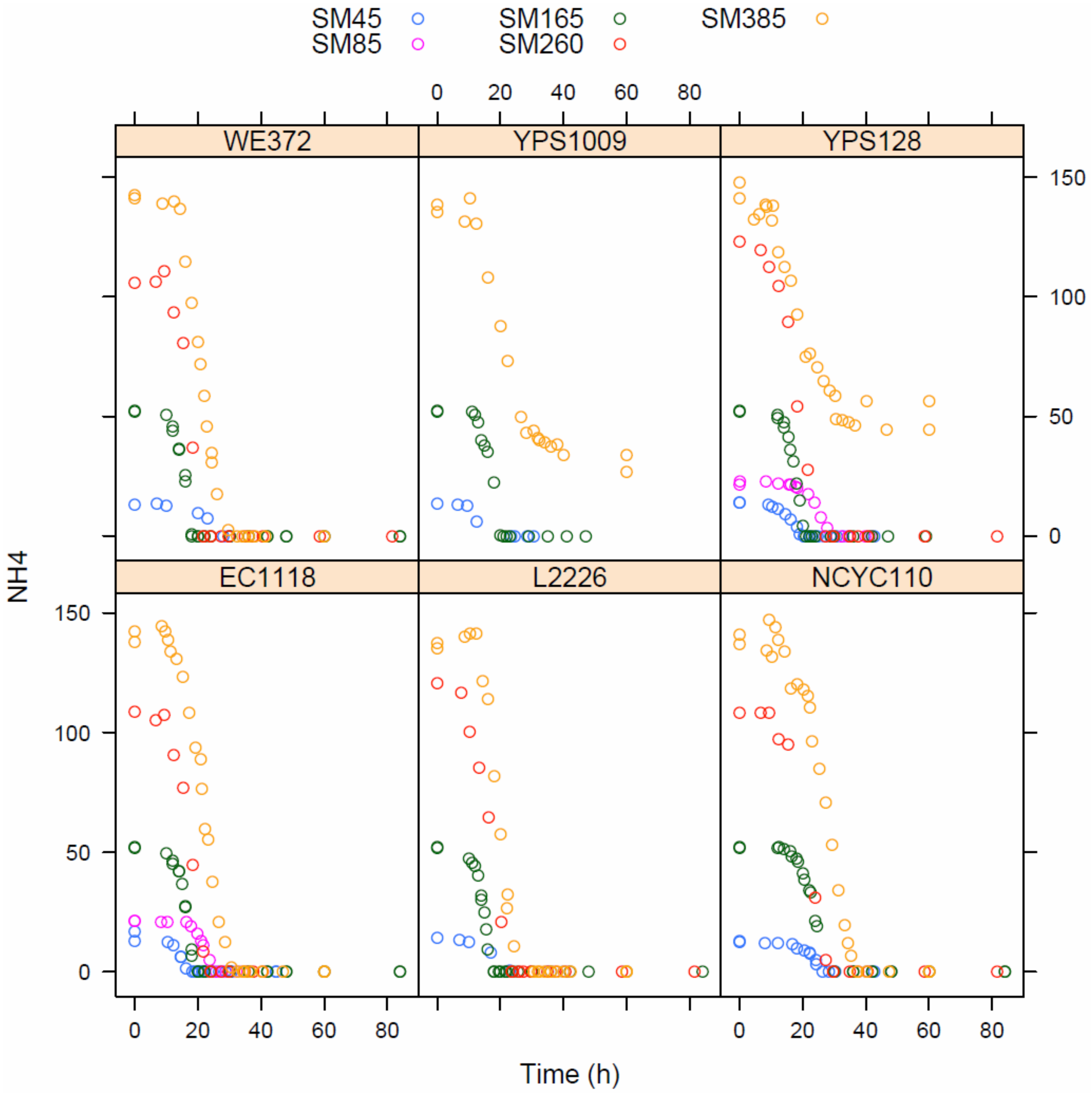
SM45
SM85
SM165
SM260
SM385
WE372
YPS1009
YPS128
EC1118
L2226
NCYC110
NH4
Time (h)
0
20
40
60
80
0
50
100
150

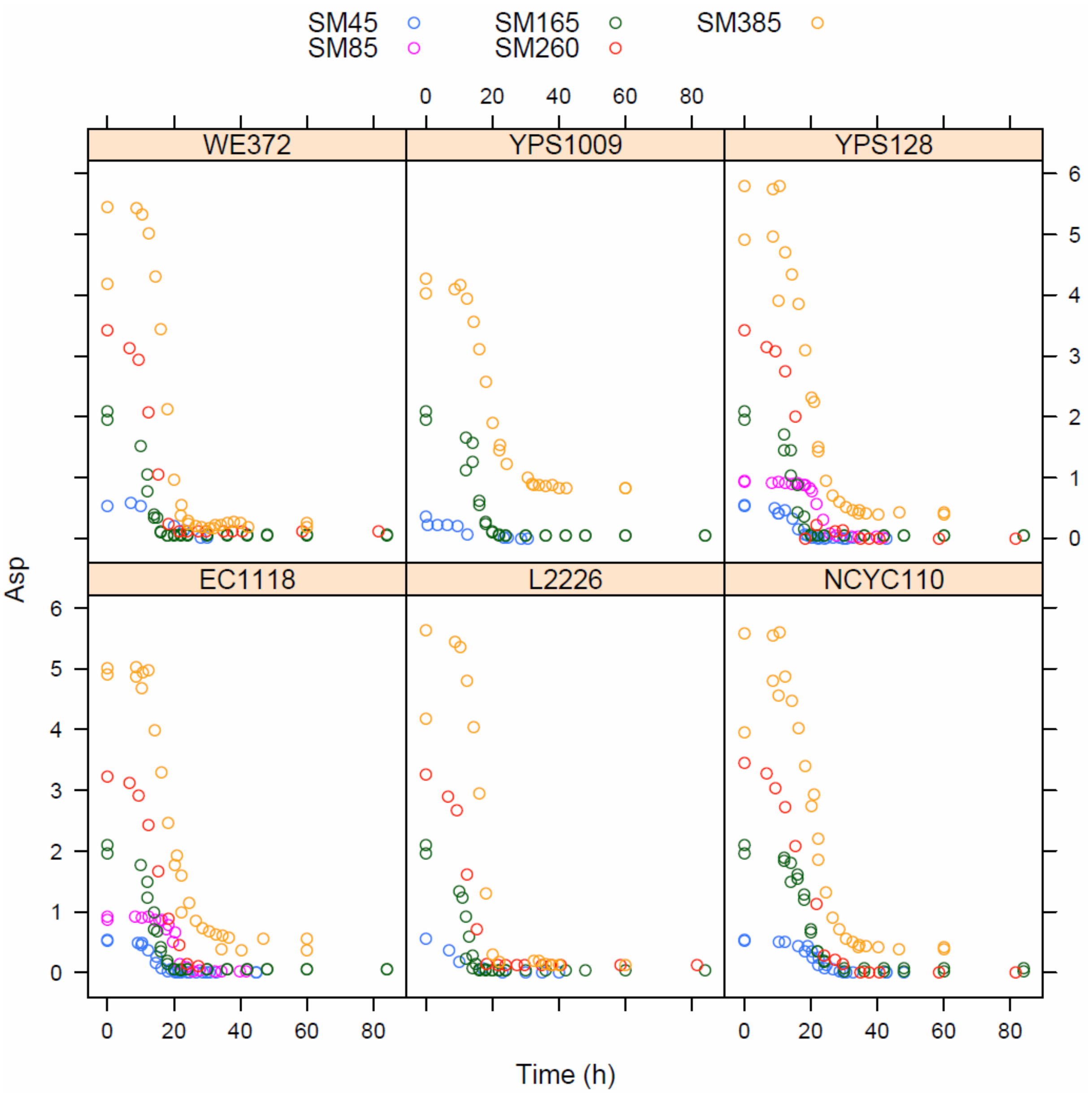
SM45
SM85
SM165
SM260
SM385
WE372
YPS1009
YPS128
EC1118
L2226
NCYC110
Asp
Time (h)

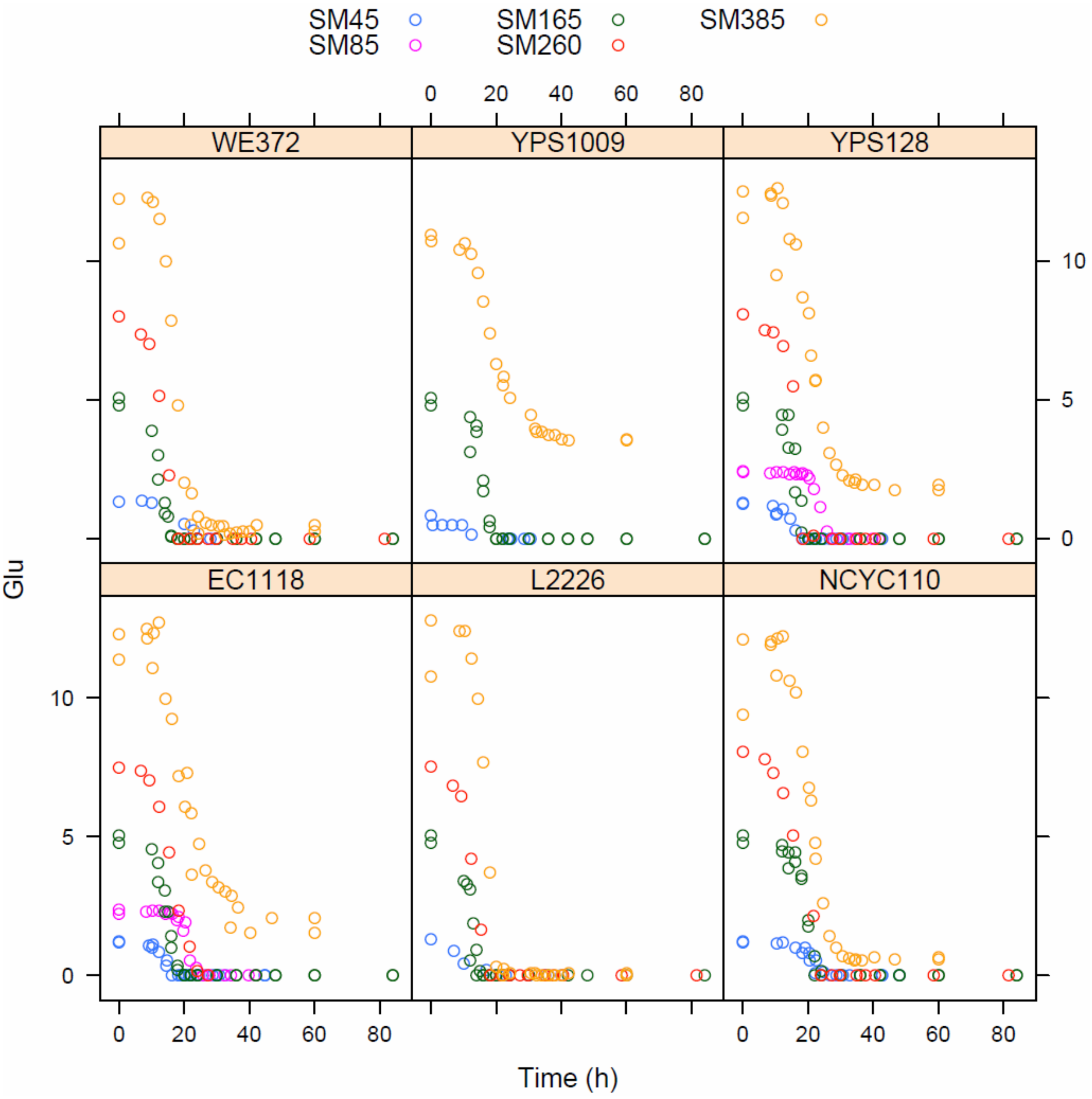

SM45
SM85
SM165
SM260
SM385
WE372
YPS1009
YPS128
EC1118
L2226
NCYC110
Glu
Time (h)
0
20
40
60
80
0
5
10

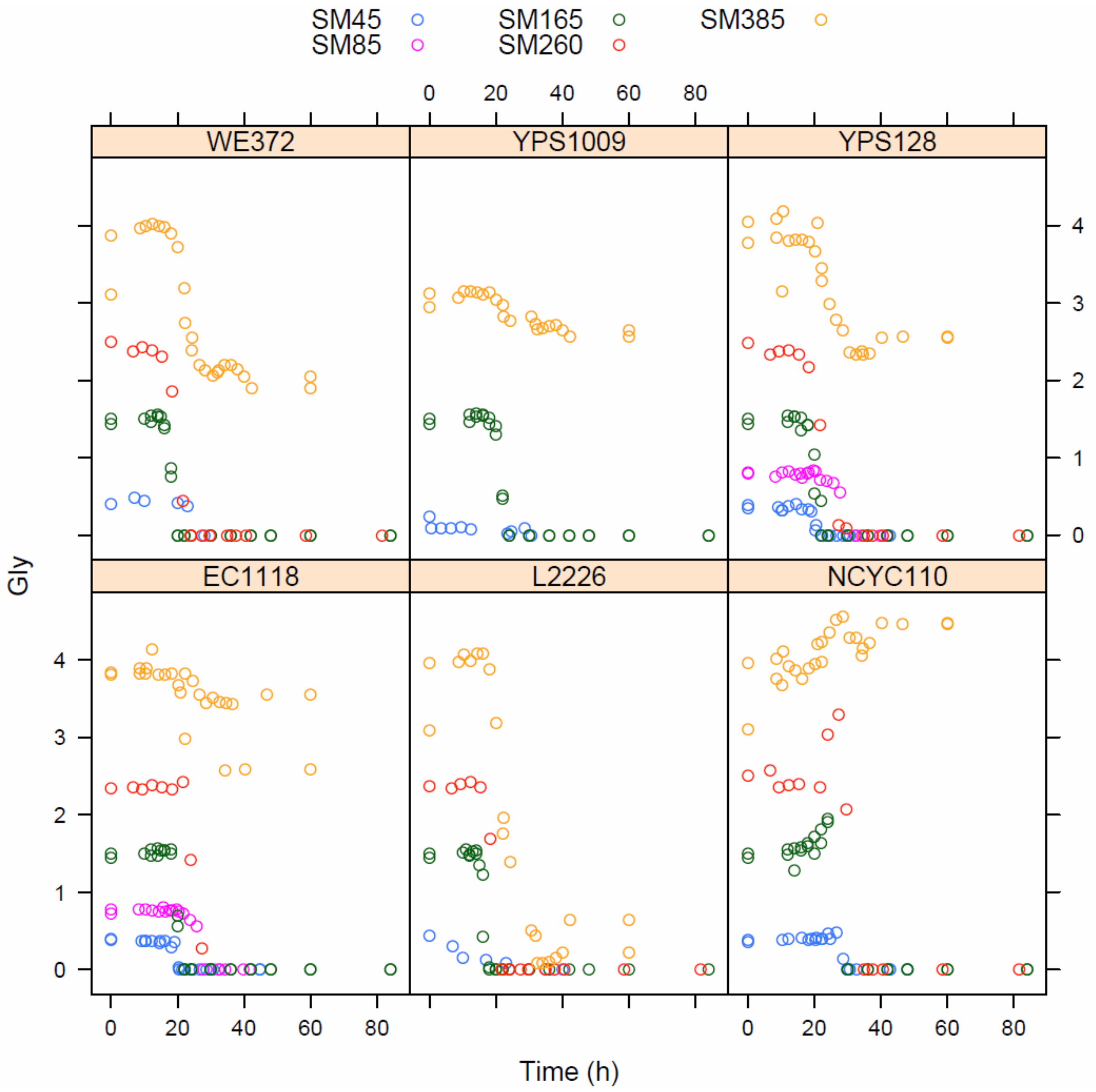

SM45
SM85
SM165
SM260
SM385
WE372
YPS1009
YPS128
EC1118
L2226
NCYC110
Gly
Time (h)
0
20
40
60
80
0
1
2
3
4

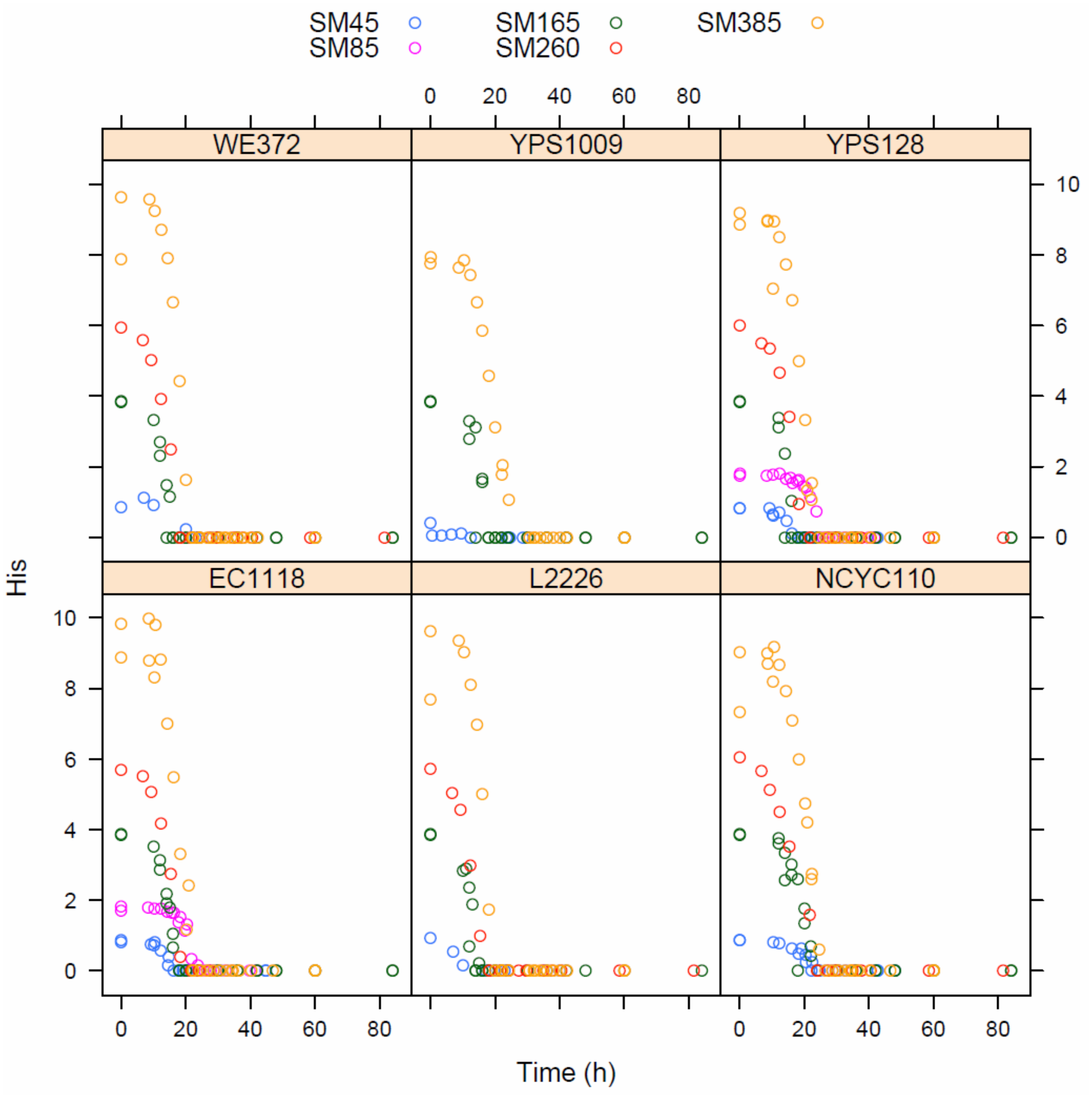
SM45
SM85
SM165
SM260
SM385
WE372
YPS1009
YPS128
EC1118
L2226
NCYC110
His
Time (h)
0
20
40
60
80
0
2
4
6
8
10

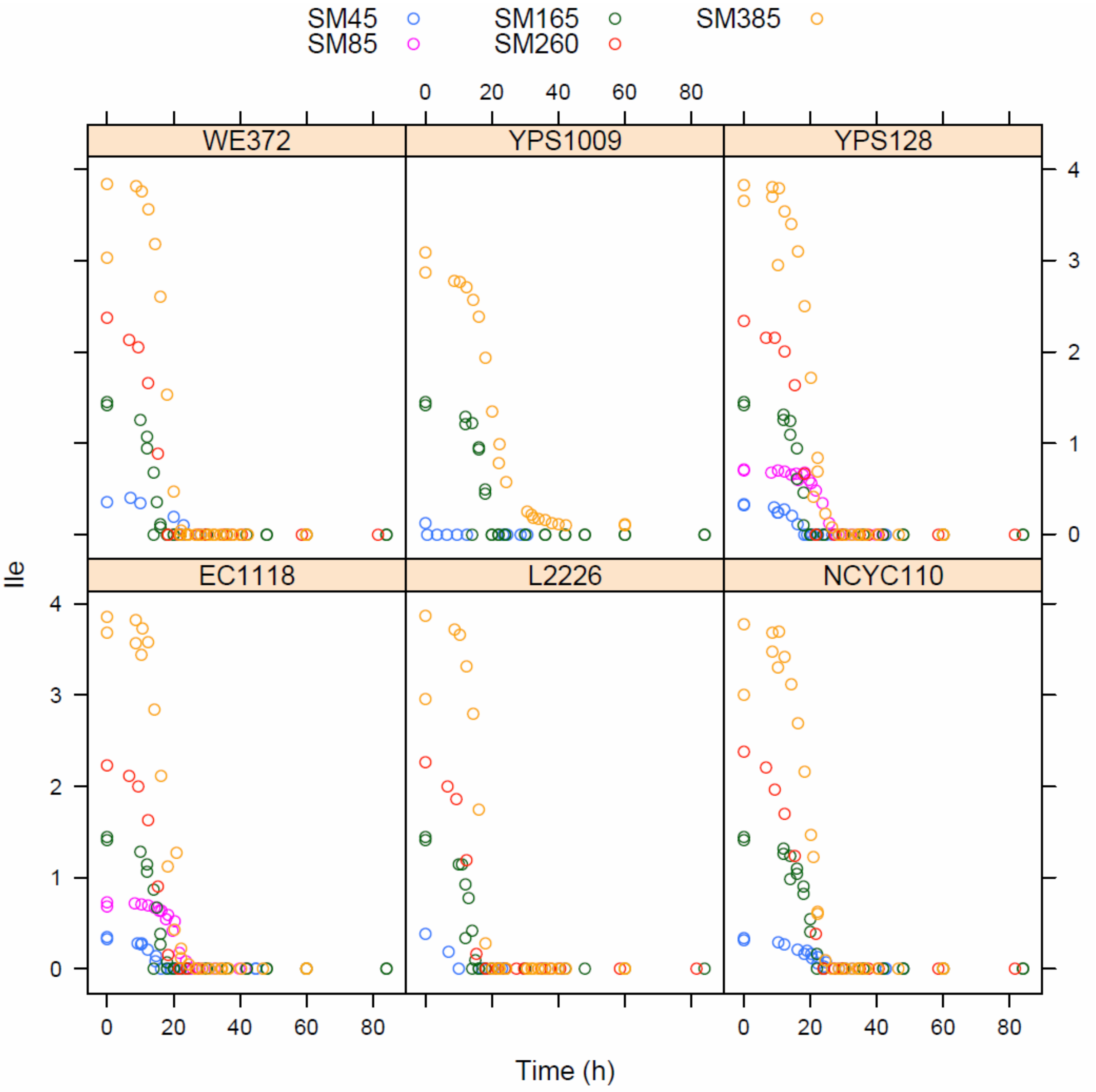

SM45
SM85
SM165
SM260
SM385
WE372
YPS1009
YPS128
EC1118
L2226
NCYC110
Ile
Time (h)
0
20
40
60
80
0
1
2
3
4

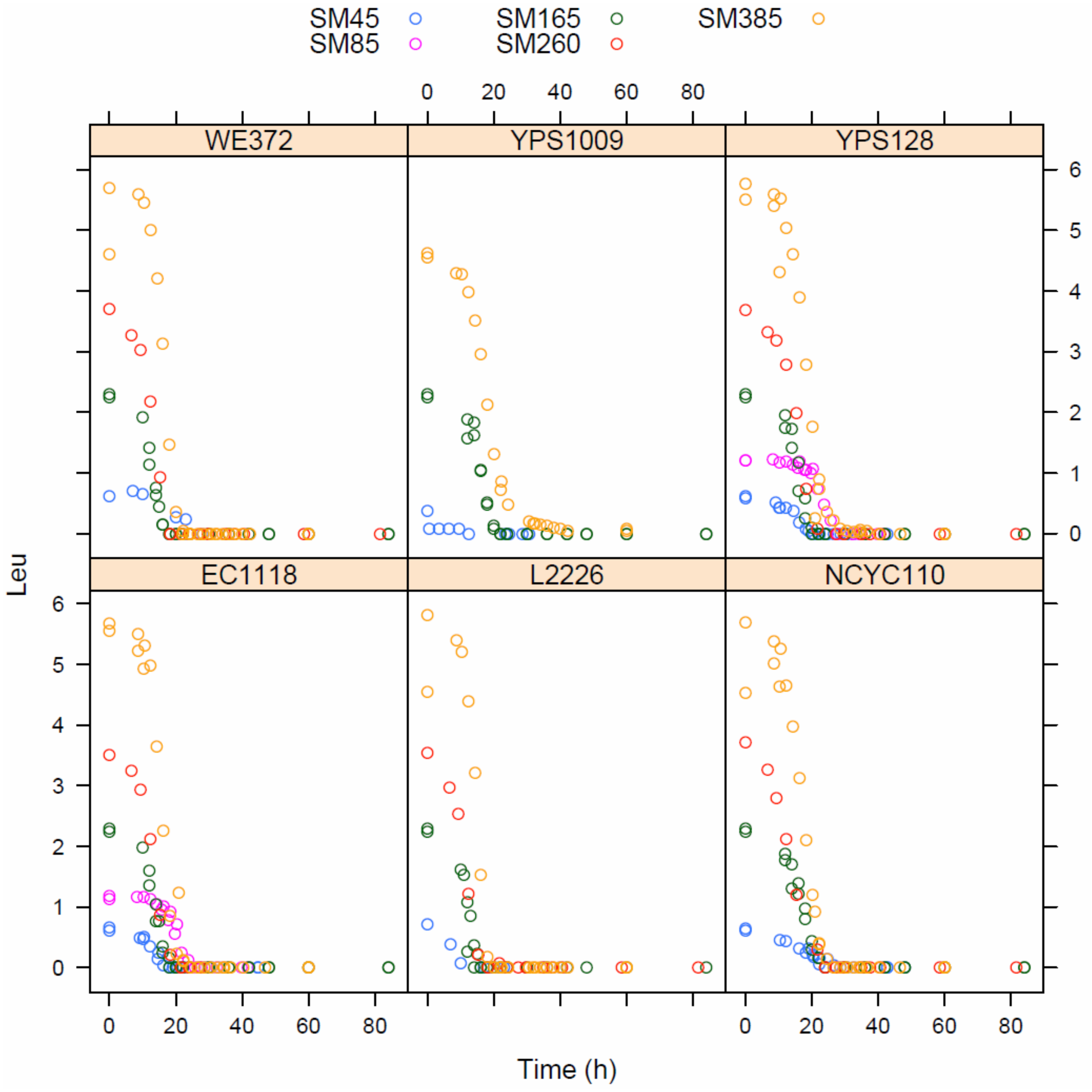

SM45
SM85
SM165
SM260
SM385
WE372
YPS1009
YPS128
EC1118
L2226
NCYC110
Leu
Time (h)
0
20
40
60
80
0
1
2
3
4
5
6

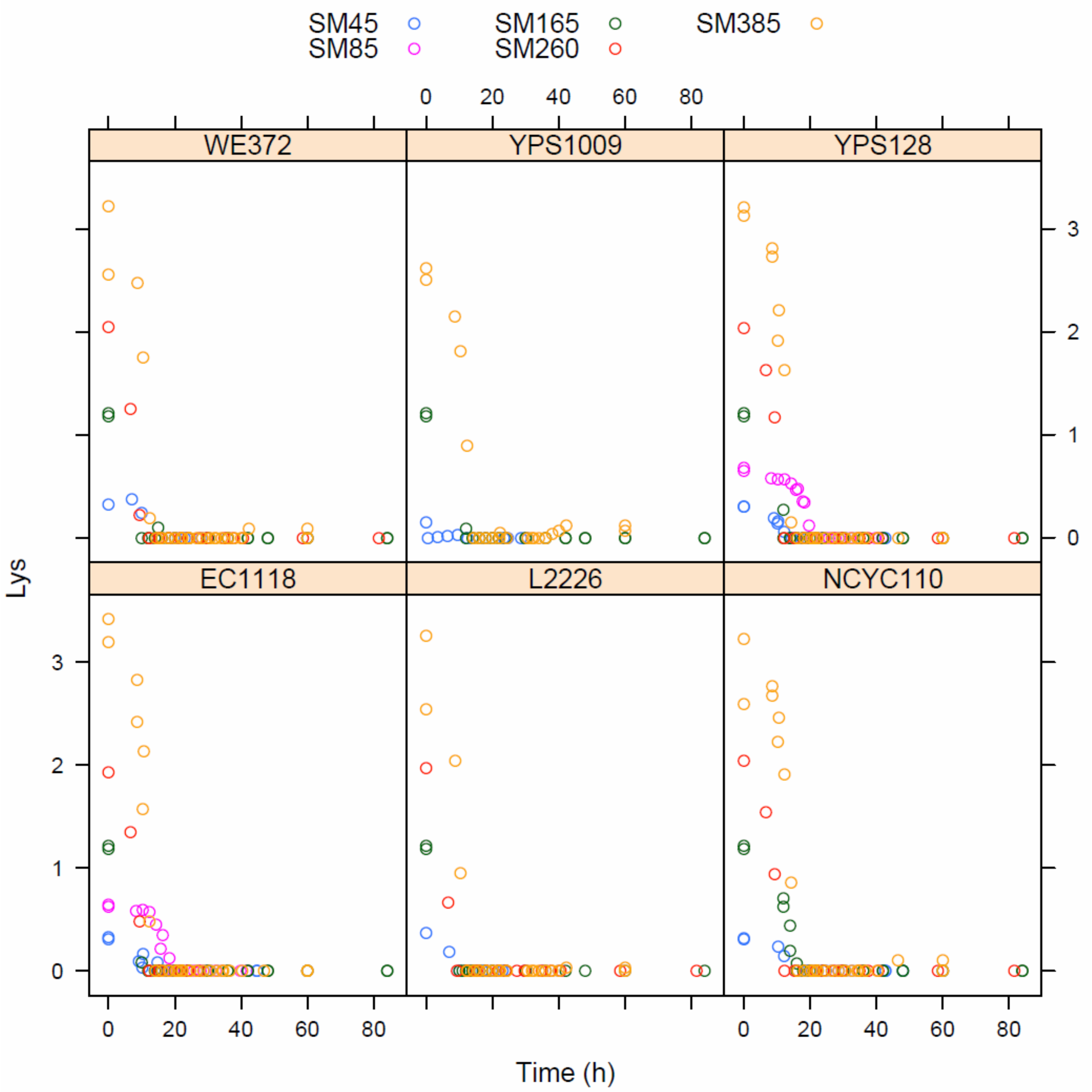

SM45
SM85
SM165
SM260
SM385
WE372
YPS1009
YPS128
EC1118
L2226
NCYC110
Lys
Time (h)
0
20
40
60
80
0
1
2
3

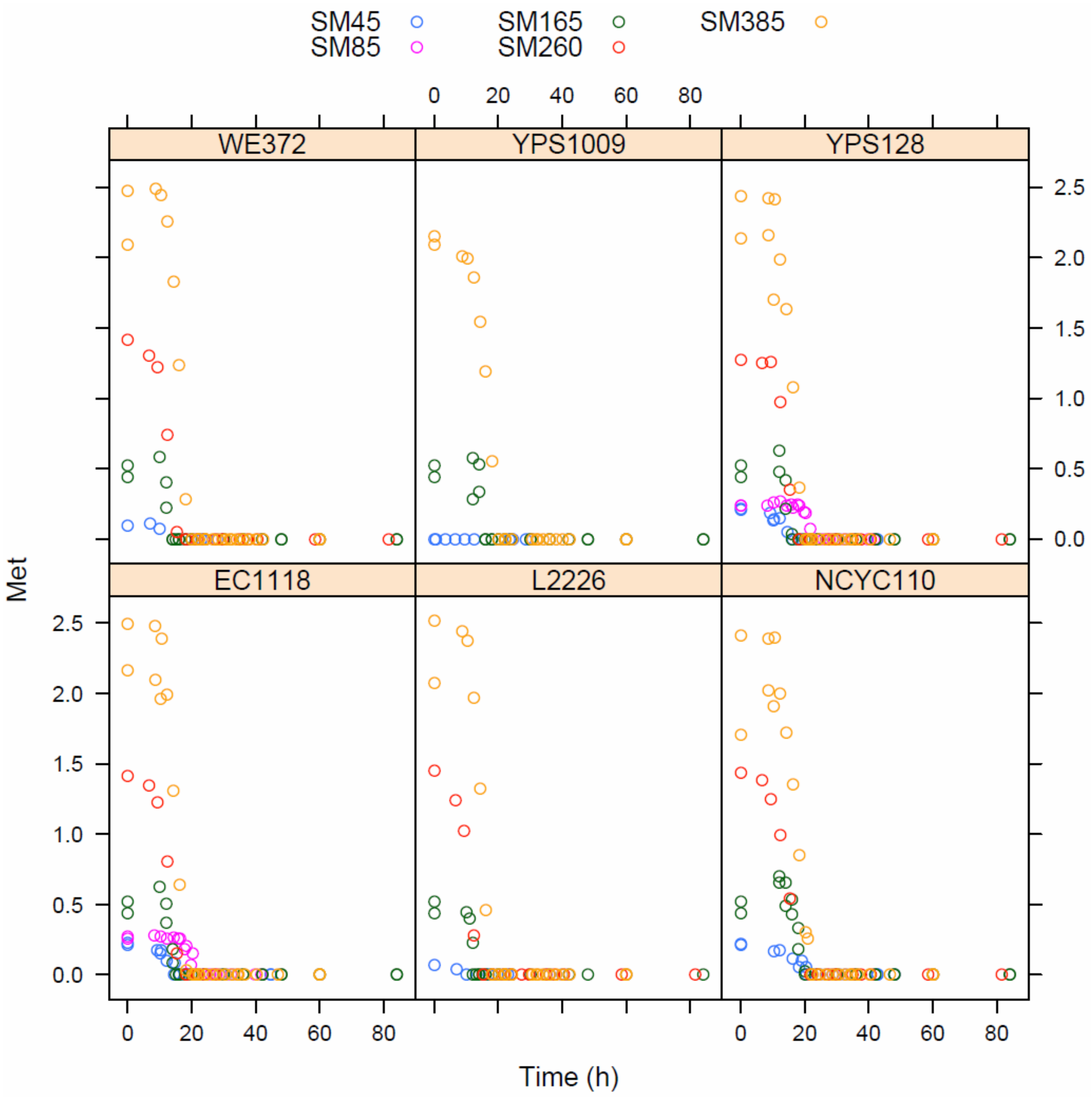

SM45
SM85
SM165
SM260
SM385
WE372
YPS1009
YPS128
EC1118
L2226
NCYC110
Met
Time (h)
0
20
40
60
80
0.0
0.5
1.0
1.5
2.0
2.5

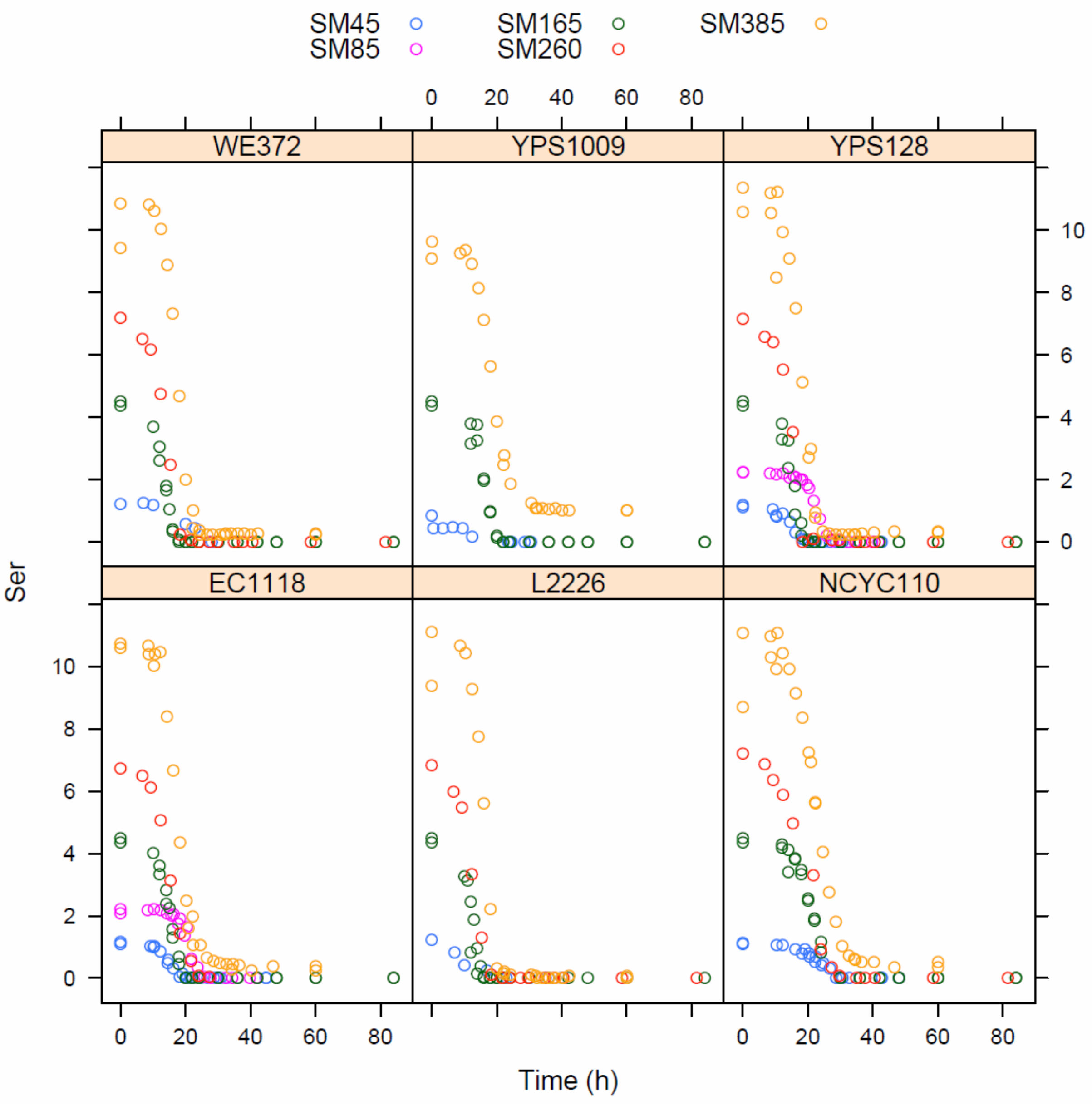

SM45
SM85
SM165
SM260
SM385
WE372
YPS1009
YPS128
EC1118
L2226
NCYC110
Ser
Time (h)

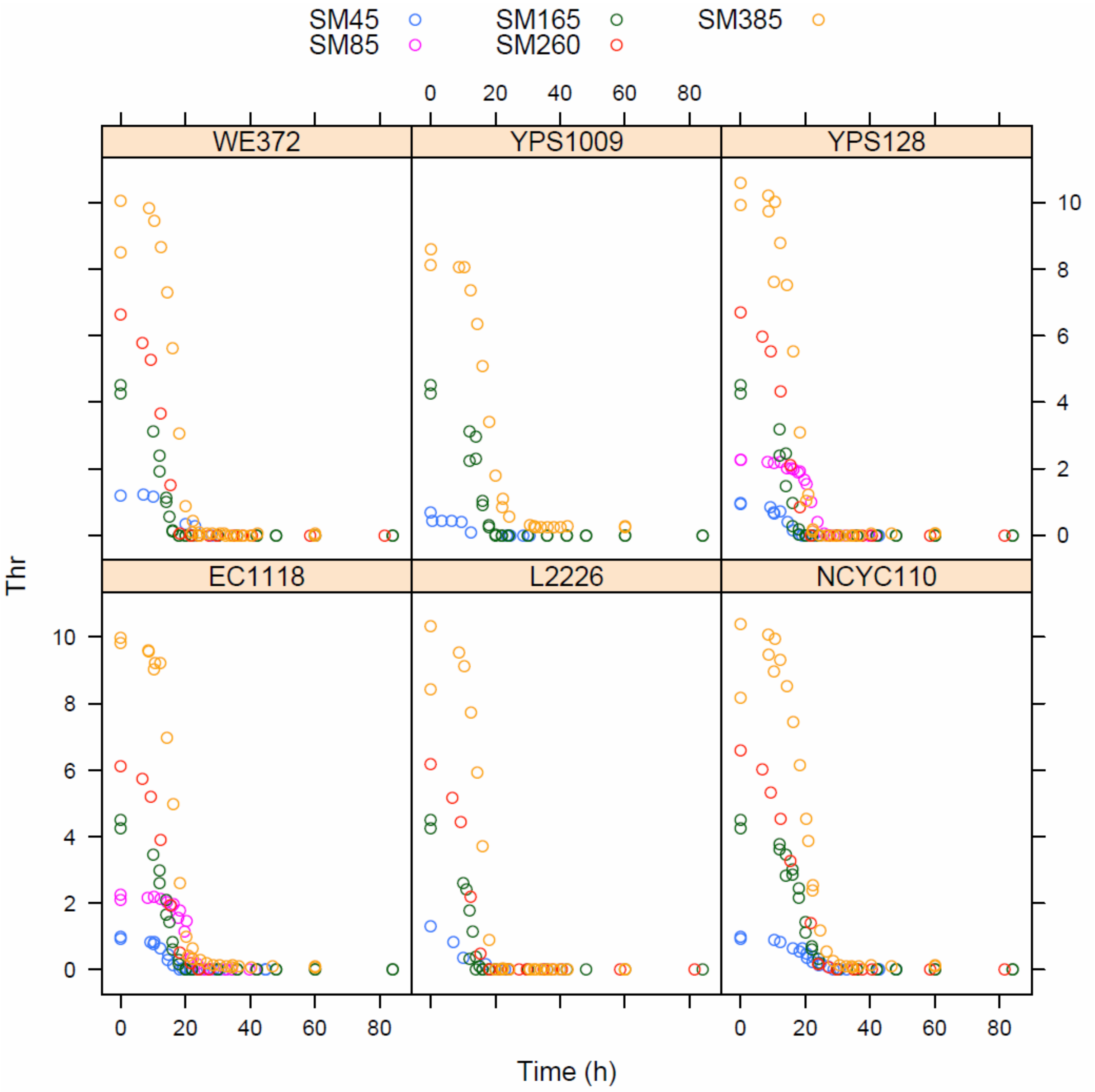
SM45
SM85
SM165
SM260
SM385
WE372
YPS1009
YPS128
EC1118
L2226
NCYC110
Thr
Time (h)
0
20
40
60
80
0
2
4
6
8
10

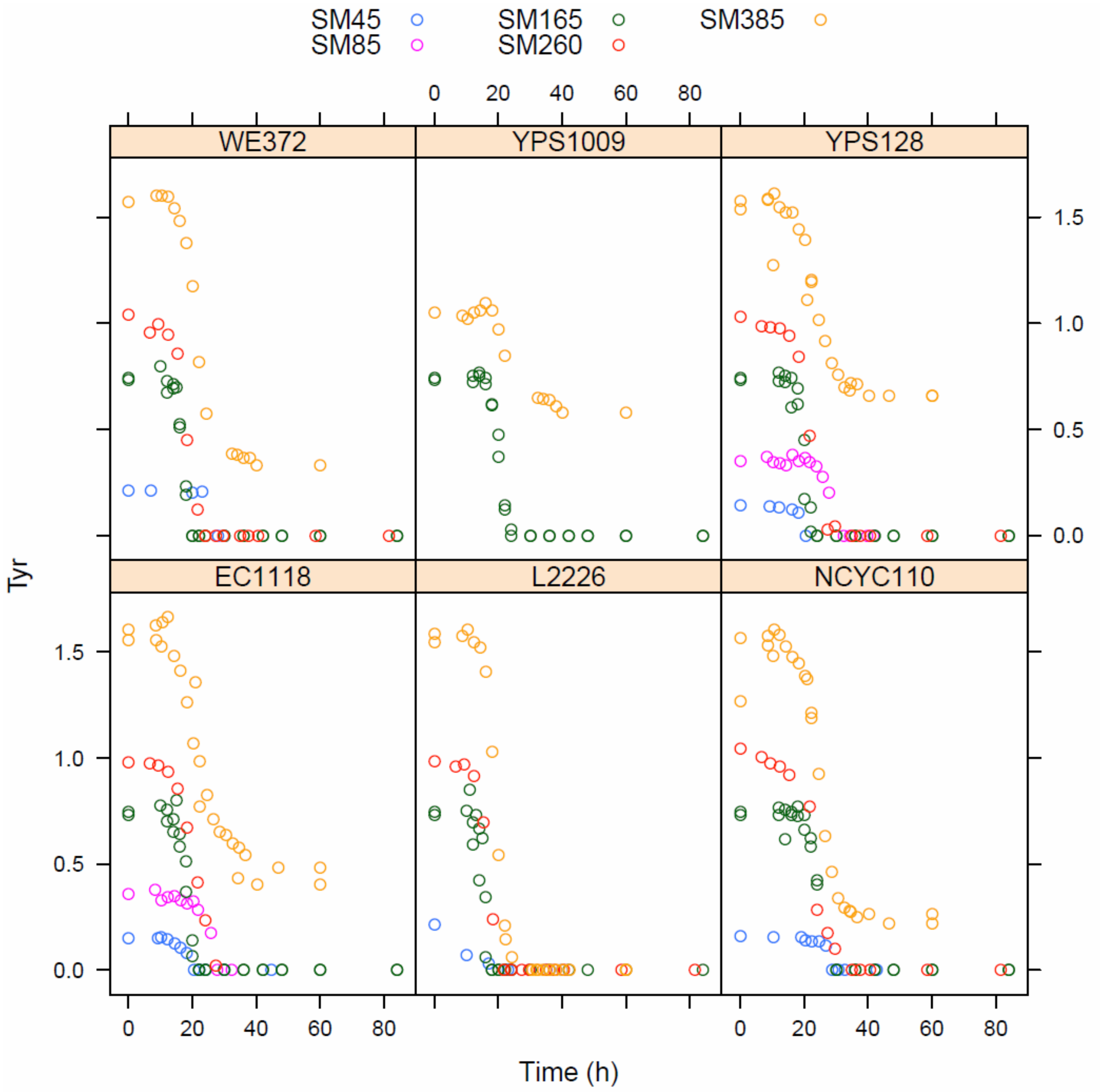
SM45
SM85
SM165
SM260
SM385
WE372
YPS1009
YPS128
EC1118
L2226
NCYC110
Tyr
Time (h)
0
20
40
60
80
0.0
0.5
1.0
1.5

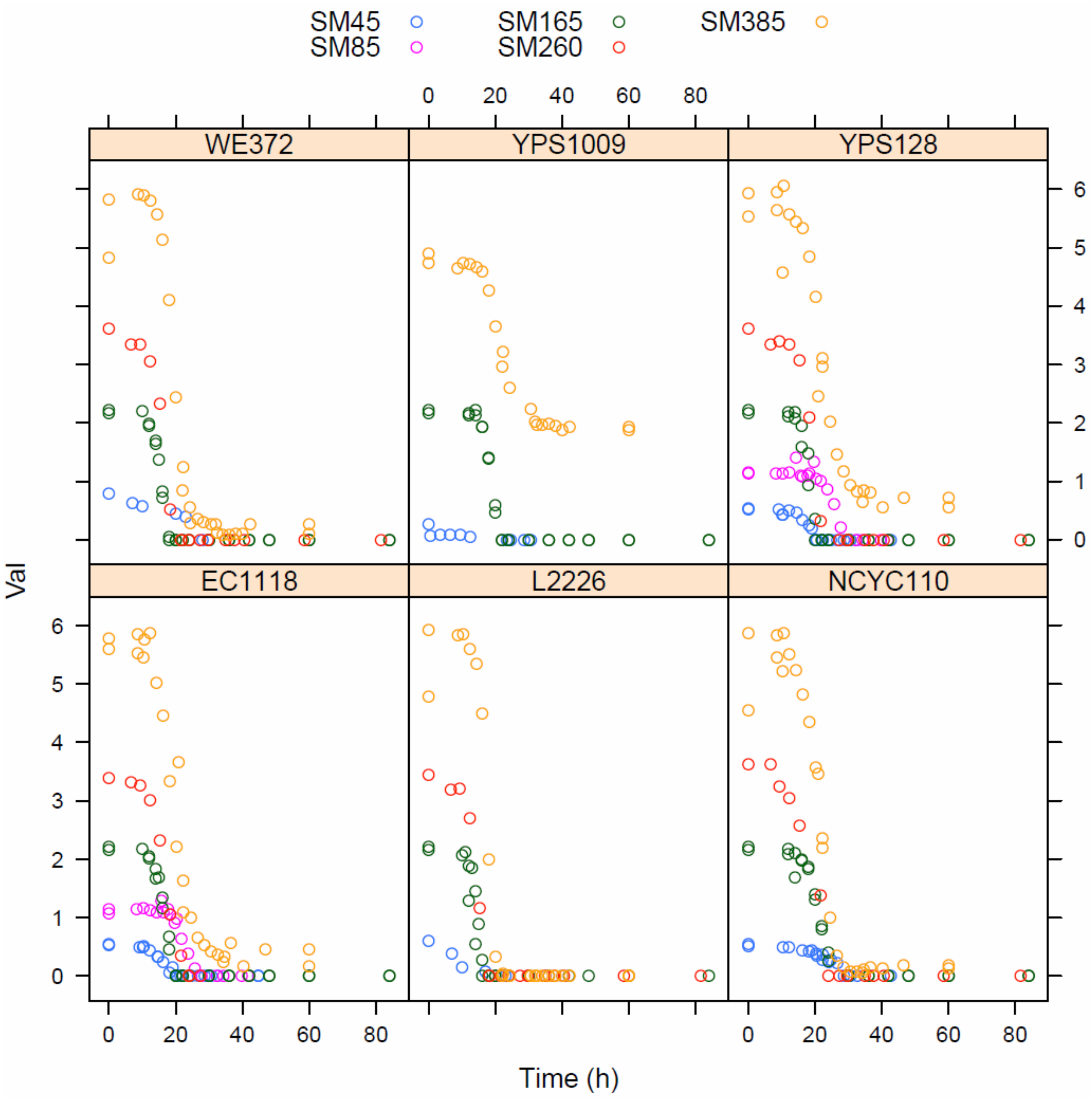
SM45
SM85
SM165
SM260
SM385
WE372
YPS1009
YPS128
EC1118
L2226
NCYC110
Val
Time (h)
0
20
40
60
80
0
1
2
3
4
5
6

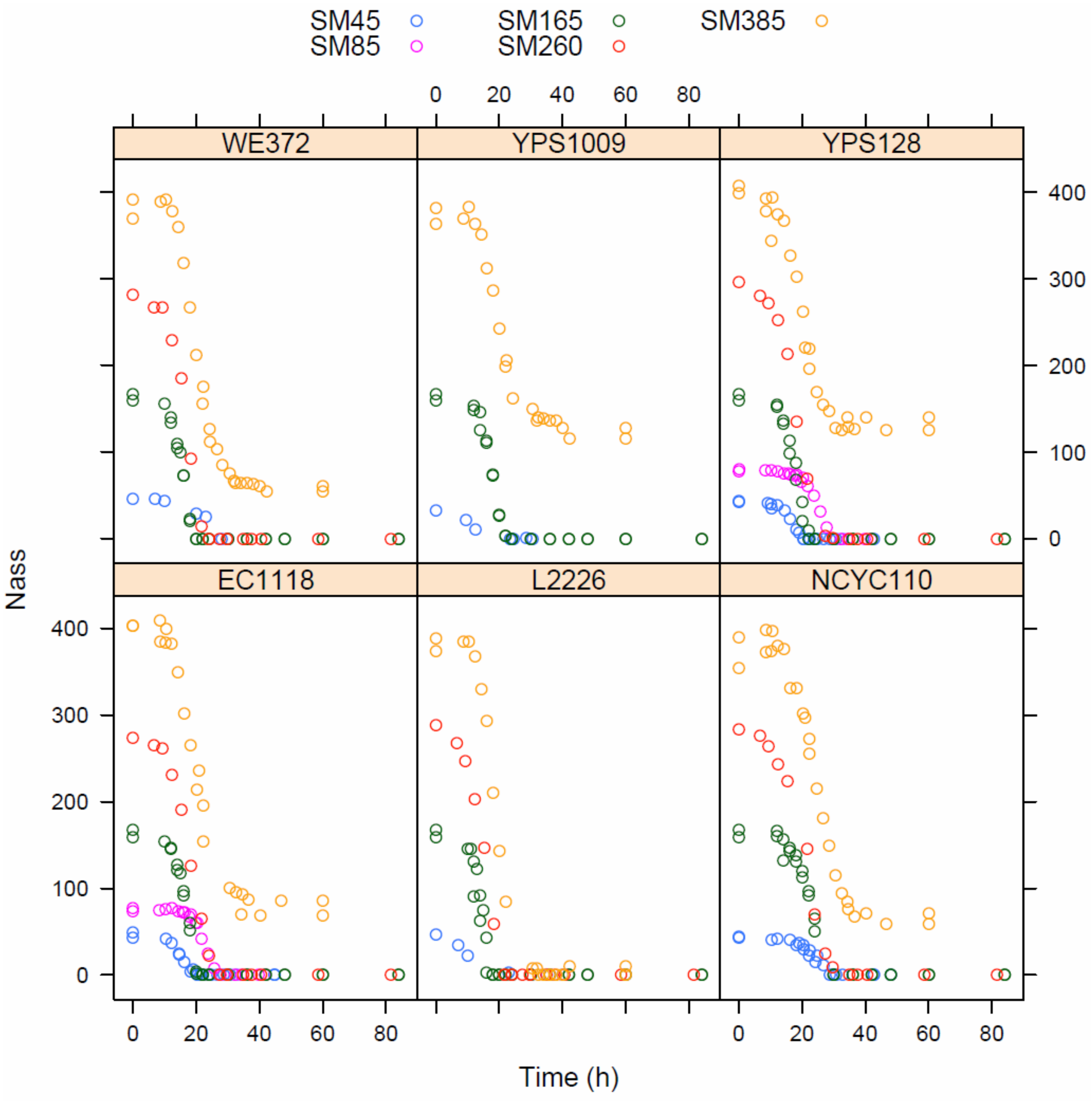

SM45
SM85
SM165
SM260
SM385
WE372
YPS1009
YPS128
EC1118
L2226
NCYC110
Nass
Time (h)
0
20
40
60
80
0
100
200
300
400
